# Growth Factors VEGF-A_165_ and FGF-2 as Multifunctional Biomolecules Governing Cell Adhesion and Proliferation

**DOI:** 10.3390/ijms22041843

**Published:** 2021-02-12

**Authors:** Antonín Sedlář, Martina Trávníčková, Roman Matějka, Šimon Pražák, Zuzana Mészáros, Pavla Bojarová, Lucie Bačáková, Vladimír Křen, Kristýna Slámová

**Affiliations:** 1 Laboratory of Biomaterials and Tissue Engineering, Institute of Physiology of the Czech Academy of Sciences, Vídeňská 1083, CZ 14220 Praha 4, Czech Republic; Antonin.Sedlar@fgu.cas.cz (A.S.); Martina.Travnickova@fgu.cas.cz (M.T.); Roman.Matejka@fgu.cas.cz or or simon.prazak@fbmi.cvut.cz (Š.P.); 2 Department of Physiology, Faculty of Science, Charles University, Viničná 7, CZ 12844 Praha 2, Czech Republic; 3 Faculty of Biomedical Engineering, Czech Technical University in Prague, CZ 27201 Kladno, Czech Republic; bojarova@biomed.cas.cz; 4 Laboratory of Biotransformation, Institute of Microbiology of the Czech Academy of Sciences, Vídeňská 1083, CZ 14220 Praha 4, Czech Republic; zuzana.meszaros@biomed.cas.cz (Z.M.); kren@biomed.cas.cz (V.K.); 5 Department of Biochemistry, University of Chemistry and Technology Prague, Technická 6, CZ 16628 Praha 6, Czech Republic

**Keywords:** heterologous expression, recombinant vascular endothelial growth factor (VEGF), basic fibroblast growth factor (bFGF), adult stem cells, endothelial cells, cell adhesion, cell proliferation, tissue engineering, regenerative medicine, vascular replacements

## Abstract

Vascular endothelial growth factor-A_165_ (VEGF-A_165_) and fibroblast growth factor-2 (FGF-2) are currently used for the functionalization of biomaterials designed for tissue engineering. We have developed a new simple method for heterologous expression and purification of VEGF-A_165_ and FGF-2 in the yeast expression system of *Pichia pastoris*. The biological activity of the growth factors was assessed in cultures of human and porcine adipose tissue-derived stem cells (ADSCs) and human umbilical vein endothelial cells (HUVECs). When added into the culture medium, VEGF-A_165_ stimulated proliferation only in HUVECs, while FGF-2 stimulated the proliferation of both cell types. A similar effect was achieved when the growth factors were pre-adsorbed to polystyrene wells. The effect of our recombinant growth factors was slightly lower than that of commercially available factors, which was attributed to the presence of some impurities. The stimulatory effect of the VEGF-A_165_ on cell adhesion was rather weak, especially in ADSCs. FGF-2 was a potent stimulator of the adhesion of ADSCs but had no to negative effect on the adhesion of HUVECs. In sum, FGF-2 and VEGF-A_165_ have diverse effects on the behavior of different cell types, which maybe utilized in tissue engineering.

## 1. Introduction

Vascular endothelial growth factor A (VEGF-A) is a heparin-binding dimeric protein belonging to the VEGF gene family together with VEGF-B, VEGF-C, VEGF-D, and the placental growth factor. All these factors differ in their affinity for three VEGF receptors, i.e., VEGFR-1, VEGFR-2, and VEGFR-3. VEGF-A binds to VEGFR-1 and VEGFR-2. Among all members of the VEGF family, VEGF-A is most strongly associated with angiogenesis and acts as a signaling protein and as a growth factor promoting specific functions in vascular endothelial cells. It occurs in nine isoforms, namely VEGF_121_, VEGF_145_, VEGF_148_, VEGF_162_, VEGF_165_, VEGF_165b_, VEGF_183_, VEGF_189_, and VEGF_206_, which are generated by alternative exon splicing of the human VEGF-A gene and differ in the number of amino acids in their chains. The VEGF-A_165_ isoform is most abundantly expressed and since it plays a key role in enhancing cell proliferation and angiogenesis [1], it is used in commercially available growth media for endothelial cells, or in experimentally-designed media inducing vasculogenesis and differentiation of stem cells towards endothelial cells [2]. VEGF-A_165_ has been used in clinical trials for therapy of refractory coronary artery disease, where it was applied in the form of a gene construct [3], in the form of mRNA [4], or in the form of the protein [5]. Other clinical applications of VEGF-A_165_ (and VEGF in general) include treatment of periodontitis and reparation of jaw bone defects, where this factor has been applied as a component of a concentrated growth factor (CGF) fibrin, i.e., a new generation of platelet concentrate biomaterial, based on autologous fibrin with multiple concentrated growth factors [6]. Recent preclinical applications of VEGF-A_165_ include therapy of spinal cord injury in a rat model [7], regeneration of sciatic nerve in rats, where VEGF-A_165_ was combined with FGF-2 [8], functionalization of artificial blood vessel replacements for promoting spontaneous endothelialization of these grafts in an ovine model [9], treatment of placental insufficiency [10], and healing of cutaneous wounds in mice [11]. VEGF-A_165_ has also been widely used for experimental work in vitro, e.g., creation of vascularized tissue-engineered constructs, intended for bone regeneration [12] or functionalization of a decellularized pericardial matrix, intended for cardiovascular tissue engineering, in order to promote its recellularization with endothelial and stem cells [13]. In this context, it is worth mentioning that VEGF-A_165_ immobilized on cell cultivation substrates can also act as an extracellular adhesion molecule, binding to integrin adhesion receptors on cells [14,15].

Fibroblast growth factor-2 (FGF-2); also known as a basic fibroblast growth factor (bFGF), is a signaling molecule of the family of fibroblast growth factors. It regulates a wide range of biological processes, e.g., cell proliferation, migration, or differentiation [16]. Similar to VEGF-A_165_, it is commonly used as a supplement in commercially available media for endothelial cell growth and expansion. FGF-2 is also a common additive to growth media for adipose tissue-derived stem cells (ADSCs). It maintains the stem cell phenotype of ADSCs, enhances their proliferation, and thus it is suitable for ADSC expansion and maintenance of their therapeutic potential [17]. In addition, it is a component of media for the expansion of various cell types, e.g., chondrocytes [18] or induced pluripotent stem cells [19]. FGF-2 has been widely applied in clinical practice, particularly in cutaneous wound healing, such as second-degree burns and chronic ulcers [20,21], and in bone regenerative therapies, such as treatment of bone fractures, osteotomies, and osteonecrosis (for a review, see [22]), or treatment of periodontitis with intrabony defects ([23]; for a review, see [24]). FGF-2 also proved its efficacy in the treatment of oral lichen planus [25] or vitiligo [26]. Animal studies also proved that FGF-2 is promising for regenerative therapy of spinal cord injury [27], of liver injury and liver diseases [28], or for treatment of alopecia [29]. It also enhanced the tendon-to-bone healing in a rabbit model [30]. In studies in vitro, FGF-2 has also been shown to promote the adhesion of various cell types, including endothelial cells and ADSCs [31,32]. Immobilization of VEGF-A_165_ and FGF-2 to the surface of various synthetic and nature-derived biomaterials enhanced adhesion and proliferation of various cell types, mainly of endothelial cells, and improved the biocompatibility of these materials [33,34,35,36]. Together with VEGF-A_165_, FGF-2 has been recently used for functionalization of a fibrin/heparin coating on the inner surface of an ePTFE vascular prosthesis to promote its endothelialization [37].

VEGF-A_165_ and FGF-2 are challenging proteins to be expressed in microbial expression systems. Although they are commercially available, their prices are rather inhibitory, namely for larger experiments. When these proteins are produced in *Escherichia coli*, the formation of inclusion bodies and the necessary protein refolding are inconvenient and result in low yields of the active growth factors [38,39,40]. In addition, purification of the growth factors from an *E. coli* lysate requires total removal of bacterial endotoxins [41], which is extremely important for any in cellulo or ex vivo experiments. Therefore, we aimed to develop a simple and generally accessible method for heterologous expression and purification of native VEGF-A_165_ and FGF-2 in a eukaryotic expression system in methylotrophic yeast *Pichia pastoris*, which is generally more suitable for the expression of eukaryotic proteins [42,43]. VEGF-A_165_ has been produced in *P. pastoris* previously, however, the produced protein was fused with hydrophobin, and the biological activity of this chimeric protein was not verified [44].

Here, we describe a facile and high-yielding method for the extracellular expression of VEGF-A_165_ and FGF-2 in *P. pastoris* KM71H, followed by a single purification step to obtain proteins of sufficient quantity and quality even for large-scale experiments. Moreover, the respective proteins were also expressed with the N-terminal 8 amino acid substrate sequence for Factor XIIIa (NQEQVSPL), which is useful for covalent attachment of the growth factors into a fibrin network used for coating of cardiovascular prostheses [45]. The functionality of recombinant growth factors was verified and quantified in vitro by evaluating the metabolic activity and the cell number of human and porcine ADSCs and human umbilical vein endothelial cells (HUVECs) cultivated in media containing the newly produced growth factors. The effect of substrate-bound growth factors on cell adhesion and proliferation was evaluated as well because these factors are often used for immobilization on various biomaterials intended for tissue engineering.

## 2. Results and Discussion

### 2.1. Expression and Purification of Vascular Endothelial Growth Factor (VEGF)-A_165_ and Fibroblast Growth Factor (FGF)-2M Growth Factors

Due to the increasing number of biological research studies employing human cells that require supplementation by human growth factors, such as VEGF-A_165_ and FGF-2, efficient production of these factors has become a challenge to reduce the costs of the experiments. The major advantage of the yeast expression system (*Pichia pastoris*) is a high-yielding production of the target protein, which is secreted into the culture media, thus facilitating a simple one-step purification by, e.g., ion-exchange chromatography [42,43].

For the efficient expression of human FGF-2, its sequence was slightly genetically modified as it naturally comprises two LysArg dibasic cleavage sites recognized by the Kex2 protease important for the processing of extracellularly targeted proteins [46]. These sites had to be removed by mutagenesis (R31K/R129K), where two arginine residues were replaced by lysines to maintain the basic character. The resulting protein has been designated FGF-2M (for respective sequences see the Supplementary Materials). Moreover, both VEGF-A_165_ and FGF-2M were also designed and expressed with the N-terminal 8 amino acid substrate sequence for Factor XIIIa (NQEQVSPL), which can be used for covalent attachment of the growth factors into a fibrin network used for coating cardiovascular prostheses or implants [45].

The genes of the respective growth factors were obtained by commercial synthesis and cloned into the yeast expression vector pPICZαA via the 5′-EcoRI and 3′-KpnI restriction sites. The plasmids were electroporated into the methylotrophic expression host *Pichia pastoris* KM71H, and the transformants were selected based on zeocin resistance. The extracellular expression of the individual growth factors was screened in nutrient-rich media and the best producing clones were cryopreserved. Subsequently, the conditions for the large-scale production were explored to reach good yields of the target proteins.

VEGF-A_165_ and its variant comprising the substrate sequence for Factor XIIIa (VEGF-A_165_-FXIIIa) were expressed in the minimal media upon induction by methanol for three days. After that, the culture media were collected and the amounts of proteins in the crude media were determined (35 mg/L for VEGF-A_165_; 37 mg/L for VEGF-A_165_-FXIIIa). VEGF-A_165_ was purified from the culture media using cation exchange chromatography at pH 6.0; the final yield of the purified VEGF-A_165_ was 15 mg per 1 L of the original culture medium (43%). The multiple bands of VEGF-A_165_ probably represent different *O*-glycosylation variants of the protein (Supplementary Materials Figure S1). For the production of FGF-2M and FGF-2M-FXIIIa, the initial cultivation of *P. pastoris* cells in the nutrient-rich medium was required; then the cells were transferred into the minimal medium to facilitate the subsequent purification, and the expression of the desired proteins was induced by methanol for three days. Even under these conditions, the production was generally lower than in the case of VEGF-A_165_, e.g., 18 mg/L of FGF-2M and 15 mg/L of FGF-2M-FXIIIa were obtained from the crude media. FGF-2M was purified employing the cation exchange chromatography at pH 4.0, and the final yield of the purified protein reached 7 mg per 1 L of the original culture medium (39%; for sodium dodecyl sulfate polyacrylamide gel electrophoresis (SDS-PAGE) of the purified growth factors see Supplementary Materials Figure S1). To make the production process biotechnologically straightforward, VEGF-A_165_-FXIIIa and FGF-2M-FXIIIa were not further purified; the crude media were concentrated and used for the assay of biological activity as such. After sterilization by syringe filters, the protein solutions were supplemented with 20% (*v*/*v*) of sterile glycerol, were shock-frozen in liquid nitrogen, and stored at −80 °C without loss of biological activity.

### 2.2. Mitogenic Activity of Soluble VEGF-A_165_ and FGF-2M

#### 2.2.1. Number of Cells in Media with Growth Factors

**Figure 1:**
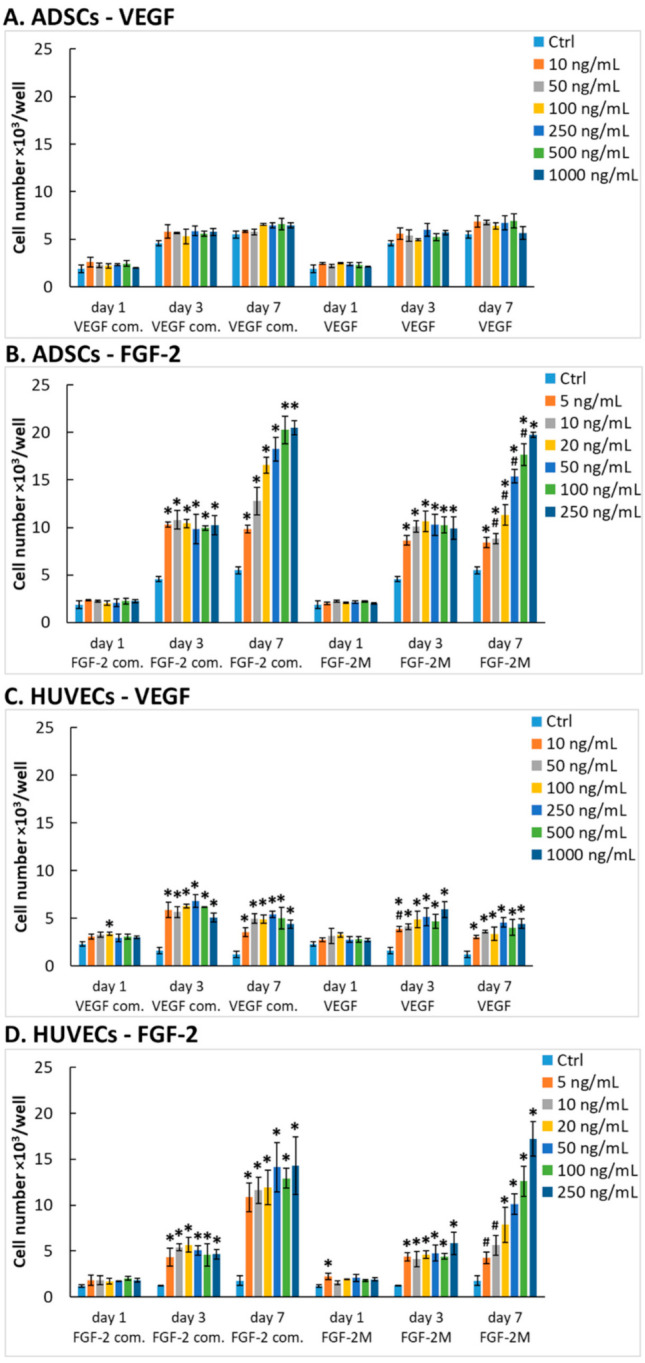
The mitogenic activity of vascular endothelial growth factor VEGF-A_165_ and fibroblast growth factor FGF-2M diluted in the culture medium. The adipose tissue-derived stem cells (ADSCs) (**A**,**B**) or human umbilical vein endothelial cells (HUVECs) (**C**,**D**) were grown in media enriched with commercial VEGF-A_165_ (VEGF com.) or our recombinant VEGF-A_165_ in concentrations from 10 to 1000 ng/mL (**A**,**C**); in media enriched with commercial FGF-2 (FGF-2 com.) or our recombinant FGF-2M in concentrations from 5 to 250 ng/mL (**B**,**D**). The growth factors were added into Dulbecco’s modified Eagle medium (DMEM) with 10% fetal bovine serum (FBS) for ADSCs (**A**,**B**), and into EGM2-weak for HUVECs (**C**,**D**). Control cells were grown in media without growth factors (Ctrl). The cell number was determined on days 1, 3, and 7 after seeding. Mean ± standard deviation (SD) from 3 wells. Holm–Sidak method, *p* ≤ 0.05. The samples were statistically compared on the indicated day after seeding. Statistically significant differences are depicted above the columns. * statistically significant difference versus control sample (Ctrl). # statistically significant difference versus sample containing a corresponding concentration of commercial growth factor.

First, we verified the mitogenic activity of our recombinant VEGF-A_165_ and FGF-2M by using these factors as supplements of the growth media for ADSCs and HUVECs. In the case of ADSCs, the mitogenic response to the two investigated growth factors markedly differed. The addition of VEGF-A_165_ in concentrations from 10 to 1000 ng/mL into the Dulbecco’s modified Eagle medium (DMEM) with 10% fetal bovine serum (FBS) did not have any significant impact on the number of ADSCs (Figure 1A). This result correlates well with a study by Khan et al. [47] where the addition of VEGF-A_165_ (50 ng/mL) into the cultivation medium did not enhance the number of ADSCs. A possible explanation is that VEGFs, in general, are endothelial-cell specific mitogens, and in different cell types, they may have different functions. Khan et al. [47] showed that VEGF-A_165_ stimulated differentiation of ADSCs towards endothelial cell phenotype, and it is known that cell differentiation is often accompanied by a decrease in cell proliferation activity. Similarly, the differentiation towards endothelial cells was observed in dental pulp stem cells exposed to a medium containing VEGF-A_165_ [2], and also in circulating monocytes adhered to tissue-engineered vascular grafts, immobilized with VEGF-A_165_ and implanted into the carotid arteries of sheep [9]. Another explanation for the insufficiency of VEGF-A_165_ to stimulate the ADSC proliferation is a lack of the VEGFR2 receptor on human ADSCs, resulting in a decreased sensitivity of these cells to VEGF, as suggested in a study by Bassaneze et al. (2010) [48]. Nevertheless, in our recent study, VEGF-A_165_ attached to a decellularized pericardium through a fibrin mesh increased its recellularization with ADSCs and subsequent endothelialization in comparison with a pericardium modified only with fibrin [13].

When ADSCs were exposed to FGF-2M in concentrations from 5 to 250 ng/mL in the same medium, they showed significantly higher cell numbers at all tested FGF-2M concentrations after 3 and 7 days of cultivation compared with the cells in the control medium without FGF-2M (Figure 1B). Moreover, on day 7, a clear positive correlation of the cell number with the FGF-2 concentration was apparent. These results are in accordance with the study by Khan et al. [47], where a simultaneous increase in both proliferation and differentiation of ADSCs were obtained when VEGF-A_165_ was combined with FGF-2. In general, our results are in line with all studies, in which FGF-2 is used for expansion of various cell types, such as mesenchymal stem cells including ADSCs [17], induced pluripotent stem cells [19] or chondrocytes [18], or for various regenerative therapies, in which the cell proliferation is needed, such as healing of cutaneous wounds [20,21], regeneration of damaged or diseased bone tissue [22,24], or treatment of vitiligo, requiring proliferation of melanocytes [26].

In the case of HUVECs, the mitogenic response to the two investigated growth factors was basically similar. On days 3 and 7 of cultivation, these cells showed increased numbers both in the medium containing VEGF-A_165_ and in the medium containing FGF-2M in comparison with the control medium without the growth factors. On day 7, this increase was more pronounced in the medium with FGF-2. In addition, similar to ADSCs, the number of HUVECs also increased proportionally to the FGF-2 concentration, especially in the medium with our recombinant FGF-2M factor. In contrast, all concentrations of the commercial and our recombinant VEGF-A_165_ increased the number of HUVECs to similar values (Figure 1C,D). Also, HUVECs treated with all the concentrations of VEGF-A_165_ showed the highest cell number on day 3, and longer cultivation (7 days) led to a slight decrease in the cell numbers. This suggests that VEGF cannot maintain a desired proliferation rate of cells for longer time periods. This confirms the hypothesis that VEGF should be used in combination with other growth factors, as it is common in commercially available media for the growth of endothelial cells, such as Endothelial Cell Growth Medium 2 (EGM2) containing the recommended growth medium-2 supplement pack (EGM2-full; see Section 3.4. in the Materials and Methods). Among other factors, this pack contains FGF-2, which proved in our study to be capable, in both commercial and our recombinant form, to boost the proliferation of both ADSCs and HUVECs in a concentration-dependent manner. This result is in line with the fact that FGF-2 is an important component of various growth media for ADSCs and endothelial cells [17,49]. Thus, our recombinant protein behaves in full accordance with its commercial counterpart.

#### 2.2.2. Comparison of Our Recombinant and Commercial Growth Factors

In cell proliferation studies, we also compared our recombinant VEGF-A_165_ and FGF-2M prepared in *P. pastoris* with commercially available VEGF-A_165_ produced in human embryonic kidney 293 (HEK 293) cells (GenScript, Cat. No. Z03073-1) and with FGF-2 produced in *E. coli* (GenScript, Cat. No. Z03116-1). From the obtained cell numbers it is clear that both commercial growth factors generally show increased mitogenic activity at lower concentrations than our recombinant VEGF-A_165_ and FGF-2M (Figure 1). As already mentioned above, our recombinant FGF-2M contained two anti-protease amino acid mutations (R31K/R129K). FGF-2 contains two heparin-binding sites, mainly formed by clusters of basic amino acids in the positions of 102–129 and 128–144 [50]. In the literature, FGF-2 containing a single mutation in the first heparin-binding site (lysine changed for neutral amino acid alanine—K129A) showed a slightly lower capacity to bind low molecular weight heparin [51]. Quadruple mutation of residues in heparin-binding sites (R118Q/K119Q/K128Q/K129Q) caused almost no change in the mitogenic activity of FGF-2 but decreased the ability to induce chemotaxis and production of urokinase-type plasminogen activator (uPA) [52]. FGF-2 deletion mutant lacking residues 27–32 remained highly mitogenic and chemotactic but failed to induce the activity of uPA [53]. From the studies published so far, it is apparent that minor changes in the amino acid sequence of the heparin-binding site or the N-terminal part of FGF-2 do not alter its mitogenic activity [52,53]. Therefore, we presume that lower mitogenic activity of our recombinant FGF-2M per mg is caused by a non-negligible amount of impurities present in the protein solution even after purification by cation exchange chromatography (see Supplementary Materials Figure S1); thus, the actual amount, especially of FGF-2M molecules, is lower in the protein solution. A similar explanation can apply for a lower mitogenic activity of our recombinant VEGF-A_165_ per mg in comparison with the activity of the commercial VEGF-A_165_ produced in HEK 293 cells. Thus, we estimated the real amount of our recombinant growth factors by densitometric measurement of the bands with the use of ImageJ software (Figure S1). According to the densitometric analysis of lines containing samples of our recombinant growth factors, the content of VEGF-A_165_ and FGF-2M corresponds approximately to 85% and 56% of the total proteins in solution, respectively.

Despite the impurities found, our recombinant growth factors were still able to significantly increase the cell number in comparison with control cells grown without these factors, as was apparent mainly in ADSCs in the medium with FGF-2M (Figure 1B) and in HUVECs in the medium with VEGF-A_165_ or FGF-2M (Figure 1C,D). Moreover, our recombinant growth factors were able to reach comparable mitogenic activity as their commercial counterparts when used in higher concentrations, i.e., 250 ng/mL of FGF-2M in the medium for ADSCs, and 50–1000 ng/mL of VEGF-A_165_ or 20–250 ng/mL of FGF-2M in the medium for HUVECs. When the densitometric measurements are taken into account, the real concentration of our recombinant growth factors in these solutions is lower by 15% in VEGF, i.e., approx. 42–850 ng/mL instead of 50–1000 ng/mL, and by 44% lower in FGF-2M, i.e., approx. 11–140 ng/mL instead of 20–250 ng/mL.

#### 2.2.3. Morphology of Cells in Media with Soluble Growth Factors

**Figure 2:**
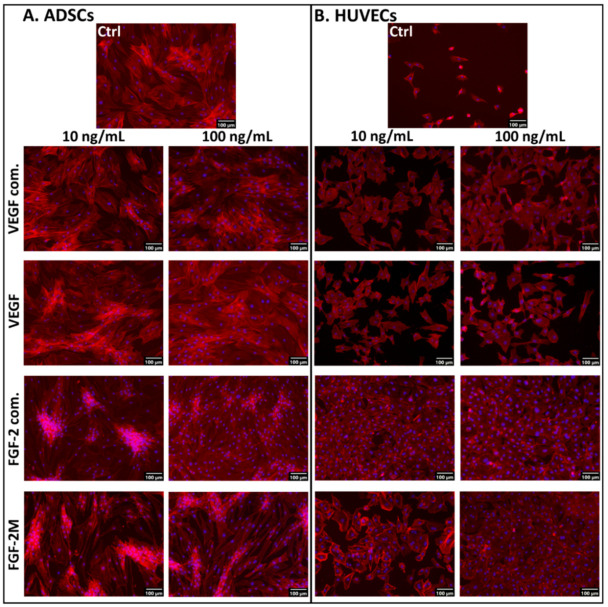
Microphotographs of ADSCs (**A**) and HUVECs (**B**) on day 7 after seeding in media enriched with commercial VEGF-A_165_ (VEGF com.) or our recombinant VEGF-A_165_, and in media enriched with commercial FGF-2 (FGF-2 com.) or our recombinant FGF-2M. The growth factors were added into DMEM with 10% FBS for ADSCs and into EGM2-weak for HUVECs. Representative low and high concentrations (i.e., 10 ng/mL and 100 ng/mL, respectively) of the tested growth factors were selected. Control cells were grown in media without growth factors (Ctrl). The filamentous actin in cells was stained with phalloidin-tetramethylrhodamine (TRITC) to visualize the cell morphology. The nuclei were counterstained with Hoechst 33258. Olympus IX 71 microscope, DP 70 digital camera, obj. 10×, scale bar 100 μm.

Our results on cell numbers were further reflected in the morphology of ADSCs and HUVECs grown in the media with VEGF-A_165_ or FGF-2M for 7 days. The ADSCs in all tested media, i.e., in the media without VEGF-A_165_, or with VEGF-A_165_ of commercial or lab-made origin, were almost confluent with similar spindle-shaped morphology, random orientation, and distribution on the cultivation substrate (Figure 2A). Similar morphology and distribution of ADSCs were also found in the medium without FGF-2, with commercially-available FGF-2 or with our recombinant FGF-2M. However, in the media with both types of FGF-2, the number of cell nuclei was apparently higher than in the control non-supplemented medium, particularly in cultures with commercial FGF-2, where the cells seemed to form multilayered clusters with cells often oriented in parallel. Interestingly, the multilayered clusters were less pronounced in higher concentrations (from 100 ng/mL) of both types of FGF-2, particularly in commercial FGF-2 (Figure 2A). 

In HUVECs cultivated in the medium without growth factors, the cells were only sparsely distributed even on day 7 after seeding, although they were well-spread and polygonal. In contrast, in the medium with commercially available or our recombinant VEGF-A_165_, the cell population density markedly increased (Figure 2B). Admittedly, the cells were not able to reach confluence at any concentration of both commercial and lab-made VEGF-A_165_. On the contrary, in the medium with commercial FGF-2, the cells reached confluence even at low FGF-2 concentrations (up to 10 ng/mL). In the medium with our recombinant FGF-2M in low concentrations, the cell population density markedly increased in comparison with the control medium, and in high concentrations (from 100 ng/mL), the cells were able to reach full confluence (Figure 2B). Taken together, the commercial FGF-2 was more efficient for obtaining the confluence of endothelial cells than our recombinant FGF-2M, which can be attributed to the fact that the real concentration of our FGF-2M in the medium is lower due to the presence of impurities in its stock solution (see above the Section 2.2.2).

**Figure 3:**
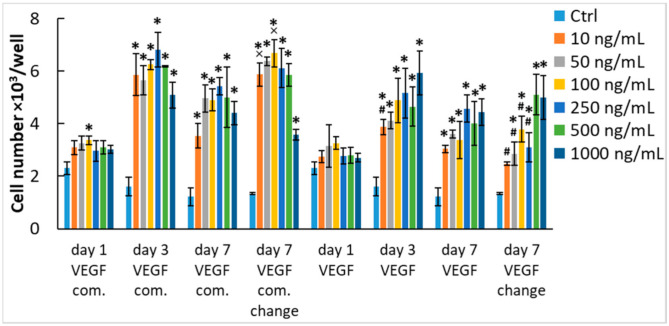
The effect of medium exchange on the mitogenic activity of VEGF-A_165_ diluted in the culture medium. The HUVECs were grown in media enriched with commercial VEGF-A_165_ (VEGF com.) or our recombinant VEGF-A_165_ in the concentration range from 10 to 1000 ng/mL. VEGF-A_165_ was added into EGM2-weak. Control cells were grown in media without growth factors (Ctrl). The cell number was determined on days 1, 3, and 7 after seeding. In some of the samples, the medium containing the corresponding concentration of growth factor was exchanged for a fresh one on day 3 after cell seeding. Mean ± SD from 3 wells. Holm–Sidak method, *p* ≤ 0.05. The samples were statistically compared on the indicated day after seeding. Statistically significant differences are depicted above the columns. * statistically significant difference versus control sample (Ctrl). # statistically significant difference versus sample containing the corresponding concentration of commercial growth factor. × statistically significant difference versus sample containing the corresponding concentration of growth factor without medium exchange.

Since the HUVECs did not reach confluence after 7 days of cultivation with both types of VEGF-A_165_ (i.e., commercial and our recombinant), and their number even decreased when compared with day 3 of cultivation (Figure 1C), we decided to perform an additional experiment. In a set of samples, we exchanged the medium on day 3 for a fresh medium with corresponding concentrations of the commercial or our recombinant growth factor (Figure 3). The results showed that the replacement of the medium with a medium with fresh growth factor maintained or even slightly improved the proliferation of HUVECs but the cell number values still did not surpass the values on day 3. This suggests that VEGF-A_165_ becomes depleted from the medium in a relatively short time period and cannot stimulate proliferation for longer incubation times, which is in contrast with FGF-2 where the cell number values were the highest on day 7 of cultivation (Figure 1D). Taken all together, it is apparent that VEGF is only a weak mitogen for endothelial cells. When expanding endothelial cells in vitro, it is important to exchange the growth medium at least twice a week and to enrich the medium also with other growth factors (e. g., FGF-2, EGF, or IGF-1) to induce rapid and continuous cell growth.

#### 2.2.4. Metabolic Activity of Cells in Media with Soluble VEGF-A_165_ and FGF-2M

The results on the cell numbers were further verified by evaluating the metabolic activity of cells, measured by the resazurin assay (Supplementary Materials Figure S2). Cell metabolic activity is generally accepted as an indirect marker of cell proliferation activity and number. We found out that in HUVECs, the cell metabolic activity correlated well with the cell numbers, corresponding also with significant differences in the cell numbers observed between the samples with our recombinant growth factors and with the commercial ones in lower concentrations. Also after the medium exchange, the numbers of HUVECs in media with VEGF-A_165_ generally corresponded to the values of cell metabolic activity (Supplementary Materials Figure S3).

However, in ADSCs, the direct cell counting and the assay of cell metabolic activity gave some different results. Interestingly, the metabolic activity of ADSCs was significantly increased by VEGF (both commercial and our recombinant) even on day 1 after seeding, while the direct cell counting did not show any differences (Figure 1A and Figure S2A). This indicates that ADSCs are responsive to this factor, although not by activating their proliferation. As mentioned above, Khan et al. [47] described the differentiation of ADSCs towards endothelial cell phenotype in a medium with VEGF-A_165_. Cell differentiation, in general, is manifested by the synthesis of phenotype-specific protein markers, which can be associated with the increased enzymatic activity of cells. Second, the metabolic activity of ADSCs in the medium with FGF-2 was almost similar to the medium with VEGF-A (Supplementary Materials Figure S2A,B), while the cell number was markedly higher in the medium with FGF-2 than in the medium with VEGF-A (Figure 1A,B). In addition, the metabolic activity of ADSCs in the FGF-2-supplemented medium was generally lower than in HUVECs (Supplementary Materials Figure S2B,D), while direct cell counting gave opposite results (Figure 1B,D). These disproportions could be explained by the fact that the resazurin assay and related assays (such as MTT, MTS, XTT, or WST—see List of Abbreviations) measure the activity of dehydrogenases in cells, which is not always linearly correlated with the cell number. The cell metabolic activity can be influenced by a wide range of factors, such as the size of cells, their nuclei and their mitochondrial area, the specific phase of the cell cycle, cell–cell contacts, 2D or 3D cultivation systems, cell population density, working volume of resazurin or incubation time. For example, in bigger cells, the activity of dehydrogenases can be relatively high at lower cell densities, while in the S-phase of the cell cycle, this activity can be relatively low [54]. The cells with relatively tight cell–cell contacts, such as cells at higher population densities or cells in 3D systems including organoids, can also show a relatively low metabolic activity, which is due to lower penetration of resazurin into the cells [55]. Besides, high cell population densities, low resazurin working volumes, or long incubation times contribute to a quick depletion of resazurin from the culture media before all cells are sufficiently stained with resorufin, a conversion product of resazurin [56].

Due to the disproportionate results on the cell number and cell metabolic activity, we decided to perform another independent and more detailed analysis of the growth of ADSCs in FGF-2-supplemented media. This analysis was also based on counting the fluorescently-stained cell nuclei, but the growth factors were added in three different concentrations, and the cells were counted in five intervals from days 1 to 7. Moreover, FGF-2M was compared not only with the commercially available FGF-2 (Genscript, Piscataway, NJ, USA, Cat. No. Z03116-1) but also with FGF-2M-FXIIIa. Moreover, the analysis was performed not only in human ADSCs but also in porcine ADSCs, i.e., in stem cells from another source widely used in experimental biomedical research.

We found that (1) all three tested forms of FGF-2 increased the number of human and porcine ADSCs in comparison with the control non-supplemented medium; (2) this increase was less apparent in both types of our recombinant FGF-2M than in the commercially-available FGF-2, except for day 7 in human ADSCs, where the effect of the highest concentration (20 ng/mL) was similar in all three forms of FGF-2; and (3) the effect of FGF-2M was usually slightly lower than in FGF-2M-FXIIIa, which was more pronounced in human than in porcine ADSCs (Supplementary Materials Figure S4). Three global trends were apparent in this data set, although some results are non-significant. First, the FGF-2 promoted cell growth in contrast to media containing only FBS. Second, there is an increased cell count with a higher concentration of all forms of FGF-2. Third, lower cell count in FGF-2M and FGF-2M-FXIIIa than in commercial FGF-2 was possibly caused by lower factor concentration due to the presence of impurities. Therefore, it can be summarized that this analysis confirmed our results on the mitogenic activity of the investigated growth factors based on the direct counting of cells on microphotographs.

### 2.3. Mitogenic Activity of Adsorbed VEGF-A_165_ and FGF-2M

#### 2.3.1. Number and Metabolic Activity of Cells on Cultivation Substrates Pre-Adsorbed with Growth Factors

In biomaterial science and tissue engineering, growth factors are often immobilized on various biomaterials to increase their bioactivity and to mimic the extracellular matrix-bound growth factors. Therefore, we evaluated whether the mitogenic activity of the studied growth factors remains preserved after adsorption on an experimental plastic surface. We measured the proliferation of ADSCs and HUVECs in wells of 96-well plates pre-adsorbed with our recombinant VEGF-A_165_ or FGF-2M in concentrations from 0.01 µM to 10 µM, which corresponded to approximately 0.192–192 µg/mL of VEGF-A_165_ and to approx. 0.172–172 µg/mL of FGF-2.

**Figure 4:**
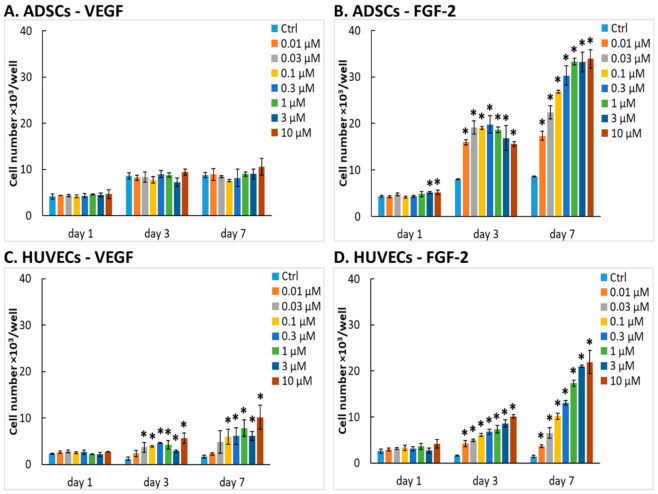
The mitogenic activity of VEGF-A_165_ or FGF-2M adsorbed on the cultivation substrate. The ADSCs (**A**,**B**) or HUVECs (**C**,**D**) were seeded in wells of 96-well polystyrene tissue culture plates pre-adsorbed with VEGF-A_165_ (**A**,**C**) or FGF-2M (**B**,**D**) in concentrations from 0.01 to 10 µM. Pristine wells without growth factors served as control substrates (Ctrl). ADSCs were grown in DMEM with 10% FBS. HUVECs were grown in EGM2-weak. The cell number was determined on days 1, 3, and 7 after seeding. Mean ± SD from 3 wells. Holm–Sidak method, *p* ≤ 0.05. The samples were statistically compared on the indicated day after seeding. * statistically significant difference in comparison with the control sample (Ctrl).

We found that the proliferation response of both cell types to the adsorbed growth factors was similar as if the growth factors were diluted in the culture media. ADSCs in wells pre-adsorbed with VEGF-A_165_ did not show almost any increase in their number and metabolic activity in comparison with control cells in the medium without the growth factor (Figure 4A and Supplementary Materials Figure S5A). Only the highest concentrations of VEGF-A_165_ (1 to 10 µM) caused a slightly elevated metabolic activity of ADSCs (Figure S5A). In contrast, the number and metabolic activity of ADSCs on immobilized FGF-2M were significantly elevated, as apparent already on day 1 after cell seeding, at least in wells with the highest FGF-2M concentrations. On day 7 after seeding, the cell numbers and metabolic activity reached the highest values at the concentrations from 1 to 10 µM (Figure 4B and Figure S5B).

In contrast to ADSCs, HUVECs displayed an increase in cell number and metabolic activity in wells pre-adsorbed with both types of growth factor, more apparently on wells pre-adsorbed with FGF-2M, especially considering the cell number. This result is rather surprising because the immobilization of VEGF to biomaterial surfaces is widely used in cardiovascular tissue engineering to enhance the growth of endothelial cells [33,34,57,58]. In these studies, the immobilized VEGF accelerated endothelialization of polymeric substrates promising for fabrication of blood vessel prostheses [33] or promoted penetration and proliferation of endothelial cells inside porous 3D collagen scaffolds in vitro [34]. Collagen scaffolds with immobilized VEGF-A_165_ were also used for the repair and vascularization of myocardial defects in rats in vivo [58]. VEGF-A_165_ in the form of a gene construct, mRNA, or protein was also clinically used for vascularization of ischemic myocardial tissue in human patients [3,4,5]. Nevertheless, in some of these cases, the effect of VEGF was further enhanced by an additional factor promoting the growth of endothelial cells, such as angiopoietin-1 [57], and particularly FGF-2 [3,5]. In a recent study, a combination of VEGF-A_165_ and FGF-2, immobilized on a fibrin mesh, was used for endothelialization of an ePTFE vascular prosthesis in vitro and is also promising for self-endothelialization of an implanted ePTFE vascular graft in vivo [37].

On FGF-2M-modified wells in our experiments, the number and metabolic activity of HUVECs clearly increased with increasing concentration of the growth factor (Figure 4C,D and Supplementary Materials Figure S5C,D). Moreover, as mentioned above, FGF-2M stimulates not only the growth of endothelial cells but also of stem cells. These cells can be used for differentiation towards various cell types used in cardiovascular tissue engineering, e.g., towards endothelial cells or vascular smooth muscle cells (for a review, see [59]). Other studies also confirmed that adsorption of FGF-2 to tissue culture polystyrene greatly enhanced the proliferation of human vascular endothelial cells [59] or human mesenchymal stem cells [60]. Similar results were also observed in studies performed on FGF-2 immobilized on non-tissue culture polystyrene, where the fetal bovine endothelial cells showed an increased proliferation rate [31,61]. In these studies, the mitogenic activity of adsorbed FGF-2 was mediated by α_v_β_3_ integrin and FGF receptor 1, and FGF-2 was expressed in *E. coli* without any mutations in its structure [31,61]. Immobilization of FGF-2 to biomaterial surfaces, such as various synthetic and nature-derived polymers, has repeatedly been proven beneficial for adhesion and proliferation of various cell types (e.g., fibroblasts, endothelial cells) [35,36,62].

It is also noteworthy that, similar to the growth factors diluted in the culture medium, the metabolic activity of HUVECs in wells with pre-adsorbed growth factors is markedly higher than in ADSCs, albeit direct cell counting gave the opposite results (Figure 4 and  Figure S5). The explanation is that the cell metabolic activity is not always linearly correlated with the cell number, although it is generally used as a marker of the cell proliferation activity (see Section 2.2.4).

#### 2.3.2. Morphology of Cells on Cultivation Substrates Pre-Adsorbed with Growth Factors

**Figure 5:**
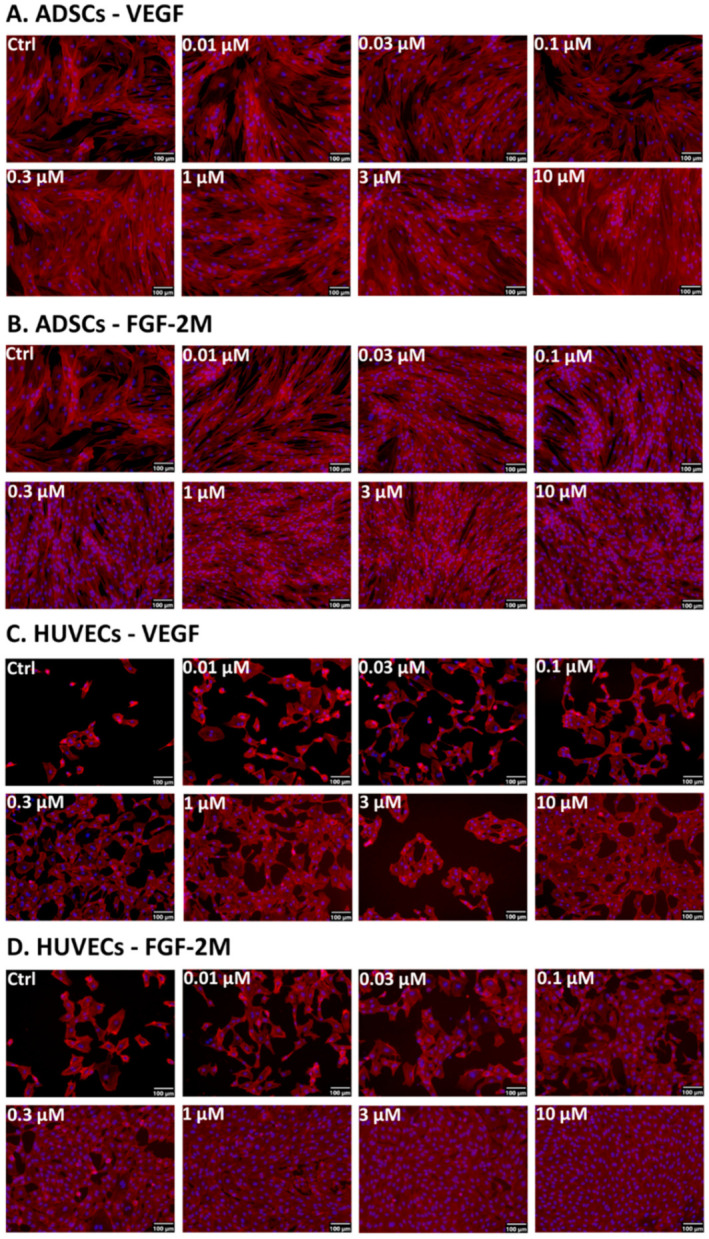
Microphotographs of ADSCs (**A**,**B**) and HUVECs (**C**,**D**) on day 7 after seeding into wells pre-adsorbed with VEGF-A_165_ (**A**,**C**) or FGF-2M (**B**,**D**) in concentrations from 0.01 to 10 µM. The filamentous actin in cells was stained with phalloidin-TRITC to visualize the cell morphology. The nuclei were counterstained with Hoechst 33258. Olympus IX 71 microscope, DP 70 digital camera, obj. 10×, scale bar 100 μm.

The morphology of cells on the substrate-bound growth factors was also generally similar to the morphology of cells grown in the media with diluted growth factors. The ADSCs cultivated on surfaces pre-adsorbed with VEGF-A_165_ or FGF-2M were mostly elongated, spindle-shaped, and randomly oriented. There was an apparent increase in the number of cell nuclei with the increasing concentration of both growth factors, especially of FGF-2M. At the highest concentrations of the adsorbed growth factors (1 to 10 µM), the cells on FGF-2M reached full confluence, while the cells on VEGF-A_165_ were rather subconfluent (Figure 5A,B).

In HUVECs, the microphotographs confirmed a generally lower cell population density than in ADSCs, especially on the surfaces pre-adsorbed with VEGF-A_165_. Even on the highest concentrations (1 to 10 µM) of VEGF-A_165_, the cells were not able to reach confluence, while on the corresponding surfaces with FGF-2M, they were fully confluent on day 7 after seeding. HUVECs on the pre-adsorbed surfaces were mostly polygonal but a considerable number of them were elongated, i.e., of a migratory and proliferative phenotype (Figure 5C,D).

### 2.4. VEGF-A_165_ and FGF-2M as Adhesion Ligands for Cells

It is known that VEGF-A_165_ and FGF-2, important growth factors, can also influence the cell adhesion when immobilized on cultivation substrates [14,32]. To investigate and compare the role of our recombinant VEGF-A_165_ and FGF-2M in the adhesion of ADSCs and HUVECs, we allowed these factors to adsorb on the bottoms of wells in 96-well E-plates, and the initial adhesion of ADSCs and HUVECs during the first 4 h after seeding was monitored in real-time using an xCELLigence sensory system.

#### 2.4.1. Adhesion of ADSCs on Cultivation Substrates Pre-Adsorbed with Growth Factors

(1) Adhesion to VEGF-A_165_

**Figure 6:**
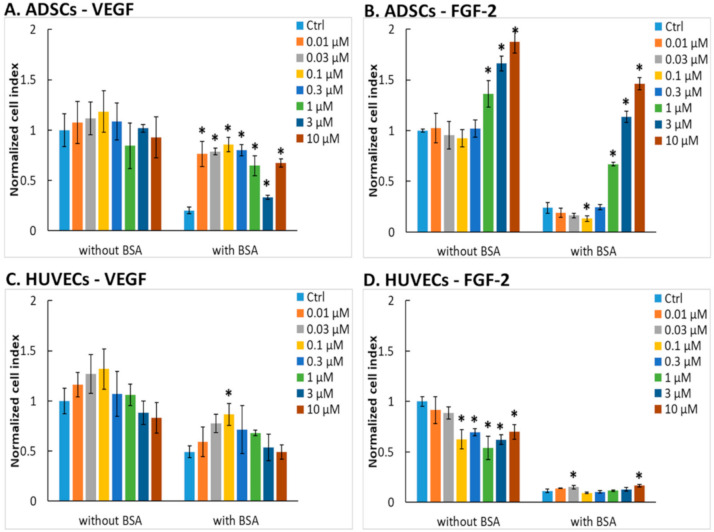
Initial adhesion of ADSCs (**A**,**B**) and HUVECs (**C**,**D**) 4 h after seeding into wells of E-plates in the xCELLIgence system pre-adsorbed with VEGF-A_165_ (**A**,**C**) or with FGF-2M (**B**,**D**) in concentrations from 0.01 to 10 µM. The wells were either left unblocked, i.e. without bovine serum albumin (BSA) or were blocked with 0.5% BSA (with BSA). Cell index values were normalized to the control sample without adsorbed growth factors and BSA (Ctrl without BSA). Mean ± SD from 3 wells. Holm-Sidak method, *p* ≤ 0.05. The statistical comparison was made amongst the samples with or without BSA. * statistically significant difference in comparison with the control sample (Ctrl).

As revealed by xCELLigence studies, the adhesion response of ADSCs to VEGF-A_165_ was similar to the growth response of ADSCs to VEGF-A_165_ in free or substrate-immobilized forms. In wells non-blocked with BSA, VEGF-A_165_ had no significant effect on the adhesion of ADSCs in comparison with control wells without the growth factor. In wells where non-specific binding sites for cells were blocked with BSA, VEGF-A_165_ slightly improved the initial adhesion of ADSCs with the optimal value at the concentration of 0.1 μM; it was significant only in comparison with wells coated with BSA alone, but not with control unmodified wells (Figure 6A). The morphology of cells was similar in all tested samples, the cells being well-spread and polygonal (Supplementary Materials Figure S6A).

The relatively low effect of VEGF-A_165_ on the adhesion of ADSCs is in accordance with previous studies by Kang et al. [32,60] who observed poor adhesion of ADSCs or other types of mesenchymal stem cell to adsorbed VEGF-A_165_. The poor adhesion-mediating ability of VEGF-A_165_ might be explained by the lack of any canonical adhesion motif, recognizable by integrin and non-integrin cell adhesion receptors in its amino acid sequence (e.g., RGD, DGEA, KQAGDV, VAPG, REDV, YIGSR, IKVAV, or KRSR; see the amino acid sequence of VEGF-A_165_ in Supplementary Materials). However, the adsorbed VEGF-A_165_ enhanced the adhesion of lymphocytic leukemia cells and B lymphocytes, mediated with the direct association of α_4_β_1_ integrin with vascular endothelial growth factor receptor-2 (VEGFR-2). The adhesion of B lymphocytes to adsorbed VEGF-A_165_ was optimal at a concentration of 8 µg/mL (0.4 µM) [15].

(2) Adhesion to FGF-2M

When the wells were pre-adsorbed with FGF-2M, the initial adhesion of ADSCs was significantly elevated at the highest concentrations of FGF-2M, i.e., from 1 to 10 μM, and increased in a concentration-dependent manner (Figure 6B). This cell response became more apparent in wells where non-specific cell adhesion sites were blocked with bovine serum albumin (BSA). In these wells, the cell adhesion to wells pre-adsorbed with 10 μM FGF-2M exceeded the value obtained in control unmodified wells, which suggested a strong affinity of ADSCs to the substrate-bound FGF-2M. These xCELLigence results were reflected by the cell morphology, especially in samples blocked with BSA. In these samples, the cells were relatively sparse and rounded in wells without FGF-2M, and their number and spreading increased with increasing FGF-2M concentration (Figure S6B). Similar results were obtained in a study by Kang et al. (2012) [32], where the adhesion of human ADSCs increased in a concentration-dependent manner up to the concentration of 10 μg/mL (ca. 0.6 µM), and then reached a plateau [32]. There, FGF-2 was co-expressed with maltose-binding protein to enhance its adsorption to a polystyrene surface. The substrate-bound FGF-2 also stimulated the adipogenic differentiation of ADSCs but not the osteogenic differentiation of these cells [32].

The positive effect of FGF-2 on the adhesion of ADSCs could be explained by the presence of two DGR sequences (positions 46–48 and 88–90), which are reverse to RGD (Arg-Gly-Asp), i.e., a well-known adhesion motif recognized by integrin adhesion receptors (see the amino acid sequence of FGF-2M in Supplementary Materials). Similarly, the DGR sequence is also recognized by integrin adhesion receptors (e.g., including α_v_β_3_), especially in its isoDGR form, which is formed by deamidation of asparagine in the NGR sequence [63,64].

#### 2.4.2. Adhesion of Human Umbilical Vein Endothelial Cells (HUVECs) on Cultivation Substrates Pre-Adsorbed with Growth Factors

(1) Adhesion to VEGF-A_165_

The adhesion response of HUVECs to VEGF-A_165_ was similar to ADSCs (Figure 6C). In wells non-blocked with BSA, the cell adhesion was improved only slightly at the intermediate VEGF-A_165_ concentration of 0.1 μM, and then it decreased again. Moreover, this improvement was statistically significant only in comparison with the highest concentration of VEGF-A_165_, but not in comparison with control wells without VEGF-A_165_ (Figure 6C). The difference between the wells without VEGF-A_165_ and those with 0.1 μM VEGF-A_165_ became significant when the non-specific cell-binding sites in wells were blocked with BSA. However, the cell adhesion in wells with VEGF-A_165_ did not surpass the control value in wells without any modification. The cells were well-spread and polygonal, but this morphology was similar in all tested samples irrespective of the presence and concentration of VEGF-A_165_ (Figure S6C). These results were rather unexpected because it is known that VEGF-A stimulates the adhesion of endothelial cells by interacting with integrin adhesion receptors on these cells [14]. However, not all isoforms of VEGF-A can bind the cells through these receptors. As mentioned above, VEGF-A is expressed in nine splice variants encoded by a single gene. These VEGF-A isoforms differ in the length of their amino acid chains, i.e., in the number of amino acids in their chains [1]. The shorter isoforms, i.e., variants with 121, 145, and 165 amino acids, are mainly diffusible, whereas the longer ones, i.e., with 189 and 206 amino acids, are sequestered in the cell membranes after secretion [14]. In the mentioned study, HUVECs seeded in plastic wells pre-coated with VEGF-A_121_, VEGF-A_165,_ or the full-length or cleaved forms of VEGF-A_189_, adhered well to the VEGF-A_189_ isoforms (cleaved or full-length), moderately to VEGF-A_165_, but not at all to VEGF-A_121_. Experiments with blocking antibodies and tumstatin, an antiangiogenic peptide, revealed that in VEGF-A_165_, the cell adhesion was mediated by α_3_β_1_ and α_v_β_3_ integrins, and in VEGF-A_189_ also by other α_v_ integrins [14]. However, this adhesion was not fully complete, which was manifested by a lack of actin stress fibers in adhering cells [14]. The binding of VEGF-A_165_ to α_v_β_3_ integrin receptors of cells can be markedly improved by fusion of this growth factor with the 10^th^ type III domain of fibronectin (FNIII10). This construct was able to activate strongly and simultaneously both VEGFR-2 and the α_v_β_3_ integrin, and thus it was more efficient in mediating cell adhesion than VEGF-A_165_ or FNIII10 alone [65].

(2) Adhesion to FGF-2M

Surprisingly, the adsorbed FGF-2M suppressed in a concentration-dependent manner the adhesion of HUVECs on wells non-blocked with BSA and showed almost no interaction with these cells in wells blocked with BSA (Figure 6D). These results were reflected in the cell morphology (Figure S6). In samples non-blocked with BSA, the cells were well-spread and polygonal, but apparently, the cell population density decreased with increasing FGF-2M concentration. In samples blocked with BSA, the cells with all tested FGF-2M concentrations were less spread and mostly rounded.

The negative effect of FGF-2M on the adhesion of HUVECs is in contrast with its strong mitogenic effect on these cells. An explanation could be the presence of an RSRK sequence in the position 116–119 in the FGF-2M molecule (see the Supplementary Materials). RSRK is the reverse sequence of KRSR. KRSR is known to bind non-integrin adhesion receptors on the cell surface, namely heparan sulfate proteoglycans, and to promote the adhesion of osteoblasts but not the adhesion of other cell types, including endothelial cells [66]. However, some studies reported a positive influence of FGF-2 on the adhesion of endothelial cells [31,61], and this adhesion was mediated by the FGF-2 fragments 24–68 and 93–120 [67], which, according to our study, contains the DGR and RSRK sequences, respectively. To the best of our knowledge, the role of RSRK in the adhesion of various cell types, stimulatory or inhibitory, has not yet been described and needs to be investigated.

### 2.5. Limitation of an In Vitro Study

Translation of recombinant growth factors into clinical applications is associated with several limitations, which arise from the growth factor preparation as well as from the system, where the potency of these factors is tested. Recombinant synthesis of growth factors can suffer from insufficient yields of recombinant expression, presence of impurities, short-term stability, and low efficiency of the final product or high production costs (for a review, see [68]). The first system of choice, in which the newly prepared growth factors are tested, is usually cell culture in vitro, which precedes the tests on animal models in vivo. This approach is in accordance with the 3R principle of the ethical use of animals in a testing (i.e., replacement, reduction, refinement). The cell cultures are believed to enable the screening of a wide range of protein variants and concentrations and to save the laboratory animals, on which only the most promising results obtained in vitro can be verified. However, it is often difficult to translate the results obtained in vitro to the conditions in vivo. It is generally known that cultured cells, especially those in a conventional static culture system in serum-supplemented media, undergo phenotypic changes (e.g., changes in the spectrum, amount, and distribution of the surface receptors), which can alter their responsiveness to various stimuli, including growth factors. After establishing the method of cell cultivation in vitro, the main advantage of this system was seen in the possibility to evaluate the biological activity of various factors on a specific single cell type without the influence of the complex and less-defined environment of the whole organism. However, this endeavor could be counterproductive, because in vivo the observed reactivity to a given factor can be modulated by the presence of other adjacent or remote cell types. For example, under in vivo conditions, the growth factors in the blood are exposed to various cell types, which could modulate their effect on endothelial cells. Conversely, one cell type can be influenced simultaneously with several growth factors. Therefore, it is difficult to extrapolate a proper growth factor concentration from studies in vitro to the conditions in vivo.

Our study aimed, at least partly, to mitigate some of these limitations. Specifically, we focused on the cost-effective recombinant expression followed by a single-step purification method resulting in high yields of the obtained growth factors. In addition, modern engineering of growth factors in vitro, in general, enables improvement of several important properties of growth factors, such as their stability, half-life, binding affinity to receptors, internalization into cells, biodistribution, and tissue penetration, and it can also modulate the binding of growth factors to the extracellular matrix (ECM) [68]. The production of human recombinant factors also paves the way to the development of novel xeno-free media for the cultivation of various cell types, particularly stem cells, needed for applications in cell therapies and tissue engineering. 

In our study, we used a conventional static cells culture system with serum-supplemented media and with monocultured cells. However, the effect of our recombinant growth factors was tested on two cell types (with different results obtained for each cell type) and in a wide range of concentrations. These concentrations were inspired by the literature, where lower concentrations (5–100 ng/mL) have been usually used for studies with soluble growth factors (e.g., [2,12,15,17,18,19,47,49]), while higher concentrations (0.5 –10 µg/mL) have been used for functionalization of various biomaterials (e.g., [12,14,15,33,34,36,37]) to simulate a local concentration of growth factors bound to the natural ECM in vivo, which can act as reservoirs of these factors, enabling their continuous release and delivery to cells [68]. 

The translatability of in vitro studies to the real situation in the organism in vivo can be further improved by co-cultivation of two or more cell types, by the use of chemically defined serum-free media, including media supplemented by recombinant growth factors and other recombinant proteins, and particularly by the use of dynamic cell culture systems. These systems provide the cells with adequate mechanical stimulation similar to that to which the cells are exposed in vivo. This stimulation can also be substituted by electrical, magnetic, gravity, or ultrasound stimulation, and is important for proper cell differentiation and phenotypic maturation. In addition, the media flow in dynamic cell culture systems enables a better supply of cells with oxygen and nutrients and quick waste removal, which further improves the physiological functions of cells [69,70]. Dynamic cultivation is, therefore, indispensable in advanced tissue engineering, which aims to create replacements of damaged tissues, closely mimicking the well-functioning tissues in a healthy organism. 

## 3. Materials and Methods

### 3.1. Expression and Purification of VEGF-A_165_

The gene of the 165-amino acid splice variant of human vascular endothelial growth factor (VEGF-A_165_) was prepared synthetically (Generay, Shanghai, China); both the nucleotide and amino acid sequences are given in the Supplementary Materials. The gene was cloned into the yeast expression vector pPICZαA comprising zeocin resistance gene downstream of the α-factor-encoding DNA segment for extracellular protein targeting using 5′-KpnI and 3′- EcoRI restriction sites. Fifteen µg of plasmid DNA were linearized employing SacI (New England Biolabs, Ipswich, US) and electroporated into the competent *P. pastoris* KM71H cells prepared according to the manufacturer’s instruction manual (EasySelect Pichia Expression Kit, Invitrogen, Waltham, MA, US). The resulting transformants were grown on yeast extract peptone dextrose (YPD) plates under the pressure of zeocin for three days at 28 °C. For a screening of the production of VEGF-A_165_ in *P. pastoris* KM71H at a small-scale, a combination of BMGY (buffered glycerol complex medium) and BMMY (buffered methanol complex medium) was used. Selected colonies were inoculated into 100 mL of BMGY and incubated at 28 °C and 220× rpm overnight. Then the cultures were centrifuged (5000× rpm, 10 min, 4 °C) and the pellets were resuspended in 30 mL of BMMY. The extracellular expression of the target protein was induced by methanol (0.5% *v*/*v*); methanol was supplemented every 24 h. The cultures were shaken at 28 °C and 220× rpm for three days. On day 5 after inoculation, the cultures were tested for the presence of VEGF-A_165_ by 15% SDS-PAGE. Clones providing the highest protein production were cryopreserved at −80 °C and employed in the large-scale production of VEGF-A_165_.

For the preparative production of VEGF-A_165_, BMGH (buffered minimal glycerol medium) and BMMH (buffered minimal methanol medium) were used starting with a preculture. The cryopreserved cultures obtained from the screening (100 µL) were inoculated into 15 mL of YPD medium and incubated at 28 °C and 220× rpm for 5 h. This preculture was inoculated into 1 L of BMGH medium in 3 L Erlenmeyer flasks and cultivated overnight at 28 °C on a rotary shaker. Then the cells were collected by centrifugation (5000× rpm, 10 min, 4 °C) and resuspended in 200 mL of BMMH medium in 1 L Erlenmeyer flasks. The culture was shaken at 28 °C and 220× rpm, the expression of VEGF-A_165_ was induced by methanol (0.5% *v*/*v*) every 24 h.

On day 5 after the first inoculation, the target protein was purified using a cation exchange chromatography column (Fractogel EMD-SO^3-^, Merck, Darmstadt, Germany) connected to the Äkta Purifier chromatography system (GE Healthcare, Chicago, IL, USA). The column was equilibrated with 10 mM sodium citrate-phosphate buffer pH 6.0. The proteins were eluted with a linear gradient of 0–2 M NaCl (60 mL, 2 mL/min). The protein concentration was assayed according to Bradford [71] using Protein Assay Dye Reagent Concentrate (Bio-Rad, Watford, UK) calibrated for γ-globulin from bovine plasma (IgG, BioRad, Watford, UK). The purity of protein fractions was determined by SDS-PAGE using 15% polyacrylamide gel. The real content of our recombinant VEGF in protein fractions after purification was determined by densitometric analysis of the gels using ImageJ software. The content was determined according to the following calculation: area under the peak corresponding to VEGF in lane histogram/sum of areas under all peaks in the lane. Fractions containing VEGF-A_165_ were collected; the buffer was changed for 100 mM Tris/HCl pH 7.4 and sterilized using 0.22 µm sterile syringe filters (Carl Roth, Karlsruhe, Germany). The protein solution was aliquoted into 1.5 mL tubes, 20% (*v*/*v*) of sterile glycerol was added and aliquoted VEGF-A_165_ was shock-frozen in liquid nitrogen and stored at −80 °C.

### 3.2. Expression and Purification of FGF-2M

The expression of human fibroblast growth factor-2 (FGF-2) was performed analogously to VEGF-A_165_, in the yeast expression system of *P. pastoris* KM71H. Two potential LysArg dibasic cleavage sites for the yeast protease Kex2 were removed by replacing Arg for Lys, and the gene of R31K/R129K FGF-2, further designated FGF-2M, was optimized and synthesized commercially (Generay, Shanghai, China; for respective sequences see the Supplementary Materials). The gene was cloned into the pPICZαA expression vector (KpnI/ EcoRI), the plasmid pPICZαA-FGF-2M was electroporated into *P. pastoris* and individual colonies were screened for FGF-2M extracellular production as described for VEGF-A_165_. The most producing clones were cryopreserved at −80 °C and used for large scale production of FGF-2M.

For the large-scale production of FGF-2M, a combination of BMGY and BMMH in smaller flasks was used. Cryopreserved cells (100 µL) were inoculated into 10 mL of YPD medium and precultures were incubated for 5 h at 28 °C with vigorous shaking. After that, 2.5 mL of precultures were inoculated into 100 mL of BMGY medium in 1 L flasks and cultivated at 28 °C and 220× rpm overnight. After centrifugation (5000× rpm, 10 min, 4 °C), the pellets were resuspended in 30 mL of BMMH medium in 300 mL baffled flasks to ensure high oxygen supply. The extracellular expression of the target protein was induced by methanol (0.5% *v*/*v*) every 24 h. The cultures were shaken at 28 °C and 220× rpm for three days.

On day 5 after the first inoculation, the FGF-2M produced was purified using a cation exchange chromatography column (Fractogel EMD-SO^3-^, Merck, Darmstadt, DE) connected to the Äkta Purifier chromatography system (GE Healthcare, Chicago, IL, USA). The column was equilibrated with 10 mM sodium citrate-phosphate buffer pH 4.0. The proteins were eluted with a linear gradient of 0–2 M NaCl (60 mL, 2 mL/min). The protein concentration was assayed according to Bradford and its purity was analyzed as described for VEGF-A_165_. Fractions comprising FGF-2M were collected, re-buffered, aliquoted, and stored as described for VEGF-A_165_.

### 3.3. Expression of VEGF-A_165_-FXIIIa and FGF-2M-FXIIIa

The constructs for the expression of VEGF-A_165_-FXIIIa and FGF-2M-FXIIIa containing an additional *N*-terminal 8 amino acid substrate sequence for Factor XIIIa (NQEQVSPL) were prepared commercially (Generay, Shanghai, China) and cloned into the pPICZαA vector. The expression of these prolonged forms of the growth factors was performed in the same way as described for the native factors. The VEGF-A_165_-FXIIIa and FGF-2M-FXIIIa obtained were concentrated from the culture media without purification in 100 mM Tris/HCl pH 7.4 buffer, sterilized, aliquoted, and stored as described above.

### 3.4. Cell Models

Human adipose tissue-derived stem cells (ADSCs) were isolated from a lipoaspirate obtained by liposuction from the thigh region of a patient (woman, aged 46 years) at a negative pressure (−200 mmHg). The isolation was conducted in compliance with the tenets of the Declaration of Helsinki for experiments involving human tissues and under ethical approval issued by the Ethics Committee in “Na Bulovce” Hospital in Prague (11 June 2019). Written informed consent was obtained from the patient before the liposuction procedure. The ADSCs were isolated by a procedure described by Estes et al. [49] with minor modifications described in our previous studies [69,72,73]. The cells were expanded in Dulbecco’s modified Eagle’s Medium (DMEM, Thermo Fisher Scientific, Waltham, MA, USA) supplemented with 10% of fetal bovine serum (FBS, Thermo Fisher Scientific, Waltham, MA, USA), 40 µg/mL of gentamicin and 10 ng/mL of FGF-2 (GenScript, Piscataway, NJ, USA, Cat. No. Z03116-1). In passage 2, the cells were characterized by flow cytometry (Accuri C6 Flow Cytometer, BD Biosciences, San José, CA, USA), using antibodies against the specific cluster of differentiation (CD) markers of mesenchymal stem cells. This method revealed the presence of standard surface markers of ADSCs, namely CD105 (endoglin, 99.9%), CD90 (immunoglobulin Thy-1, 99.5%), CD73 (ecto-5′-nucleotidase, 100%) and CD29 (fibronectin receptor, 100%). At the same time, the ADSCs were negative or almost negative for CD31, also referred to as platelet-endothelial cell adhesion molecule-1, PECAM-1 (0.5%), CD34 (antigen of hematopoietic progenitor cells, 0.2%) and CD45 (protein tyrosine phosphatase receptor type C, 3.8%), which are markers of hematopoietic or endothelial cells, and also for CD146 (4.7%), referred to as melanoma cell adhesion molecule or receptor for laminin; also considered to be a marker of pericytes [69].

Porcine adipose tissue-derived stem cells (PrADSCs) were isolated from fat surgically extracted from the neck area of experimental pigs (breed Prestice black pied pigs with a weight of approximately 35–40 kg; Institute of Animal Science, Přeštice, Czech Republic) during the surgery under general anesthesia. The protocol for cell isolation was also set according to Estes et al. [49] with some modifications developed in studies focused on adipose-derived stem or stromal cells [74,75] and is described in our previous study by Matejka et al., 2020 [70]. The cells were expanded in a standard way until the 2nd passage in DMEM-F12K (Sigma-Aldrich, St. Louis, MO, USA) medium (ratio 1:1) supplemented with 10% of FBS (Sigma-Aldrich, St. Louis, MO, USA), 1% of ABAM (Antibiotic Antimycotic Solution, contains 100 units penicillin, 0.1 mg streptomycin, and 0.25 µg amphotericin B per mL of culture media, Sigma-Aldrich, St. Louis, MO, USA) and 10 ng/mL of FGF-2 (GenScript, Piscataway, NJ, USA, Cat. No. Z03116-1). Similarly, as human ADSCs, they were characterized by flow cytometry for the presence or absence of specific CD markers, namely CD105 (96–99%), CD90 (99%), and CD29 (99%). CD73 was present only in 0.3–2.6% of the cells but is known from the literature that this marker is very low or absent in porcine ADSCs, and instead of it, CD44, i.e., hyaluronan receptor, has been usually evaluated. The prADSCs were almost negative for CD34 (0.5–1%) and CD45 (1–3%) but they showed a relatively high positivity for CD31 (29–35%) [70].

For studies on the effect of our recombinant FGF-2M, the commercial FGF-2 was removed from the medium. The cells were grown until 80% confluence and then used for testing. This was done to minimize the pooling effect of FGF-2 used in cell expansion. The fat isolation from experimental pigs was approved by the Ministry of Health of the Czech Republic, reference No. MZDZ 23132/2018-4/OVZ, approval No. 37/2018 in the Institute of Clinical and Experimental Medicine. A minimal number of animals were used. All procedures described were undertaken under general anesthesia and according to ethical guidelines to minimize the pain and discomfort of the animals. The Institute of Clinical and Experimental Medicine has authorized facilities and fully equipped operating theatres for performing these animal experiments.

Human umbilical vein endothelial cells (HUVECs) were purchased from Lonza (Basel, Switzerland, Cat. No. C2517A). The cells were grown in the endothelial cell growth medium 2 (EGM2), which was prepared from the endothelial cell basal medium 2 (EBM2, PromoCell, Heidelberg, Germany, Cat. No. C-22111) supplemented with 1% of antibiotic-antimycotic solution (*v*/*v*, Sigma-Aldrich, St. Louis, MO, USA, A5955) and the growth medium-2 supplement pack (PromoCell, Heidelberg, Germany, Cat. No. C-39211) containing hydrocortisone, heparin, ascorbic acid, EGF, VEGF, IGF-1, FGF-2 and 2% of FBS. For the purpose of this study, we decided to term this medium “EGM2-full”.

### 3.5. Cell Cultivation with Recombinant Growth Factors Diluted in Culture Medium

To confirm the mitogenic activity of the newly prepared recombinant growth factors, the cells were seeded in 96-well tissue culture plates (TPP, Trasadingen, Switzerland, Cat. No. 92096) at a density of 3 × 10^3^ cells/well. ADSCs were grown in DMEM with 10% of FBS and HUVECs in EGM2 medium containing hydrocortisone, heparin, ascorbic acid, and 2% of FBS from the growth medium-2 supplement pack (PromoCell, Heidelberg, Germany, Cat. No. C-39211). However, EGF, VEGF, IGF-1, and FGF-2 that are also components of the supplement pack were not added to the medium to prevent their interference with the tested recombinant growth factors. This medium was further termed “EGM2-weak”. This cultivation medium was further enriched with our recombinant VEGF-A_165_ or FGF-2M in the range of concentrations from 10 to 1000 ng/mL or 5 to 250 ng/mL respectively, and the cells were cultivated for 1, 3, or 7 days. In selected wells, the medium was exchanged for a fresh one with a corresponding concentration of the growth factor on day 3 after cell seeding.

An additional experiment focused on the mitogenic activity of the tested growth factors was performed on human and porcine ADSCs. The cells were seeded in 24-well tissue culture plates (Jet Bio-Filtration Co., Guangzhou, China) with an initial density of 5 × 10^3^ cells per cm^2^. As a basic culture medium, the DMEM-F12K (1:1 ratio) with 10% FBS and 1% ABAM was used. This mixture was also set as a control. Then, three different types of FGF-2 (commercial FGF-2, our recombinant FGF-2M, and FGF-2M-FXIIIa) at concentrations 5, 10, and 20 ng/mL were added. The cultivation ran for 1, 2, 3, 5, and 7 days.

### 3.6. Cell Cultivation with Recombinant Growth Factors Adsorbed on Culture Wells

The mitogenic effect of the substrate-bound growth factors was determined in the 96-well polystyrene tissue culture plates pre-adsorbed overnight at 4 °C with recombinant growth factors diluted in a phosphate-buffered saline (PBS) at concentrations ranging from 0.01 to 10 μM. The wells were then washed twice in PBS. The cells were seeded at a density of 3 × 10^3^ cells/well in 200 μL of the medium designated for each cell type. ADSCs were grown in DMEM containing 10% of FBS and HUVECs in EGM2-weak for 1, 3, or 7 days.

### 3.7. Monitoring the Initial Cell Adhesion to Adsorbed VEGF-A_165_ and FGF-2M Using xCELLigence System

The cell-adhesive properties of the tested growth factors were monitored with the use of xCELLigence Real-Time Cell Analysis - Single Plate (RTCA SP) sensing device (Agilent Technologies, Waltham, MA, USA). Wells in an E-plate (E-plate view 96 PET, ACEA Biosciences, San Diego, CA, USA, Cat. No. 300600910) were adsorbed with VEGF-A_165_ or FGF-2M (0.01 to 10 μM) overnight at 4 °C in PBS. Then, the wells were incubated with 0.5% bovine serum albumin (BSA) in PBS at 37 °C for 1 h to block the non-specific binding on the well bottoms. Some of the wells were left unblocked to observe the effect of the respective growth factor on the non-specific cell adhesion to the plastic surface (i.e., mediated by other factors, such as electrostatic interactions or adsorption of cell membrane-bound adhesion-mediating proteins, e.g., fibronectin and vitronectin, to the well bottom). The cells were seeded at the density of 10^4^ cells/well in 200 μL of pure medium without any supplements (DMEM for ADSCs; EBM2 for HUVECs). The cell adhesion was monitored every 3 min for 4 h in a cell incubator at 37 °C in a humidified atmosphere containing air with 5% CO_2_.

### 3.8. Fluorescence Staining and Counting of Cells

After the cultivation of cells with free and substrate-bound growth factors, and also after monitoring the initial cell adhesion in the xCELLigence system, the cells were processed for the evaluation of their number and morphology. The cells were fixed in 4% paraformaldehyde (10 min). The cells were blocked and permeabilized in PBS containing 1% BSA and 0.1% Triton X-100 (20 min), and then in PBS with 1% Tween-20 (20 min). The cells were stained with phalloidin-TRITC (100 ng/mL in PBS; 1 h; 25 °C) to visualize the filamentous actin (F-actin), which is a component of the cell cytoskeleton. The nuclei were counterstained with Hoechst 33258 (10 μg/mL in PBS; 1 h; 25 °C). The cells were observed in the Olympus IX71 epifluorescence microscope (DP71 digital camera, objective magnification 10×). The number of cells grown with free or substrate-bound growth factors was evaluated on days 1, 3, and 7 after cell seeding by counting the cell nuclei on four randomly selected microphotographs for every well. The cell number was then presented as a mean from 3 wells for each experimental group and time interval. The morphology of initially adhering cells monitored in xCELLigence system was evaluated at the end of monitoring, i.e., 4 h after seeding.

In the additional experiment with human and porcine ADSCs, the fixed cells were counterstained with DAPI (Sigma-Aldrich, St. Louis, MO, USA). Then 10 randomly selected fields of view were taken of each sample using a Leica DMi8 epifluorescence microscope with a 5× objective and digital camera. Custom MATLAB (MathWorks, Natick, MA, USA) script was used to batch count cell nuclei at each image.

### 3.9. Metabolic Activity Assay

The metabolic activity of ADSCs and HUVECs cultured in 96-well plates was measured with a resazurin assay. Resazurin (Sigma-Aldrich, St. Louis, MO, USA, Cat. No. R7017) is a non-toxic redox indicator. In metabolically active cells, intracellular reductases reduce resazurin to fluorescent resorufin, and this fluorescence is then detected spectrophotometrically. The cell metabolic activity was determined on days 1, 3, and 7 after seeding. The stock solution of resazurin in PBS (4 mM) was diluted to the final concentration of 40 μM in the fresh cultivation medium (DMEM without phenol red containing 10% FBS for ADSCs and EGM2-weak for HUVECs). The cells were incubated for 4 h at 37 °C in a medium containing resazurin (0.1 mL/well). Fluorescence (Ex/Em = 530/590 nm) was measured on Synergy™ HT Multi-Mode Microplate reader (BioTek, Winooski, VT, USA). Every sample was measured in triplicate. Resazurin solution in a medium without cells was used as a background control. The cell metabolic activity was regarded as an indirect marker of the cell proliferation and the cell number.

### 3.10. Statistical Analysis

In experiments determining the cell number (Figure 1, Figure 3 and Figure 4), the cell index (Figure 6) and the cell metabolic activity (Figures S2, S3 and S5), the data are presented in bar graphs as mean ± standard deviation (SD) from 3 wells. The samples were statistically compared using one-way analysis of variance (ANOVA), Holm-Sidak method. The statistical comparison was carried out using SigmaPlot 14.0 software (Systat Software Inc., San Jose, CA, USA). In Figure S4, showing the influence of FGF-2 on the growth of human and porcine ADSCs, the data are presented as mean ± SD from 10 randomly selected fields of view, and the statistical significance was evaluated using non-parametric Kruskal–Wallis one-way ANOVA on ranks, Dunn’s method, in MATLAB. In all used methods of statistics, the value of *p* ≤ 0.05 was considered significant.

## 4. Conclusions

Our study presents a new and cost-effective method for extracellular expression of VEGF-A_165_ and FGF-2 in a eukaryotic system of *P. pastoris*. FGF-2 was produced as a double mutant termed FGF-2M (R31K/R129K) to avoid degradation by Kex2 proteases. When added to the culture media (VEGF-A_165_: 10–1000 ng/mL, FGF-2: 5–250 ng/mL), both recombinant growth factors showed mitogenic activity, which was stronger in FGF-2; this factor supported the growth of both ADSCs and HUVECs, while VEGF-A_165_ supported the growth of HUVECs only. When the factors were adsorbed to a plastic surface in concentrations from 0.01 μM to 10 μM (corresponding to approximately 0.192–192 µg/mL for VEGF-A_165_ and 0.172–172 µg/mL for FGF-2), their mitogenic activity remained unaltered. The mitogenic activity of our recombinant growth factors was slightly lower than that of their commercially available counterparts, which can be explained by the presence of some impurities, and thus by a lower actual concentration of the growth factors in the solution. Furthermore, the effect of the adsorbed growth factors on the initial adhesion of ADSCs and HUVECs was determined. Both cell types, especially ADSCs, showed a relatively low affinity to adsorbed VEGF-A_165_. Interestingly, recombinant FGF-2 enhanced the adhesion of ADSCs but not the adhesion of HUVECs, which even tended to decrease with increasing FGF-2 concentration. Our results suggest that the coating of the biomaterial surface with VEGF-A_165_ and FGF-2 can direct the adhesion and growth of different cell types in different ways. This knowledge can be utilized in regenerative medicine, particularly in tissue engineering, where the cell adhesion, growth and further differentiation should be modulated by a controllable manner. 

## Data Availability

The data presented in this study are available on request from the corresponding authors.

## Supplementary Materials

The following are available online at https://www.mdpi.com/1422-0067/22/4/1843/s1, Figure S1: Amino acid and nucleotide sequences of growth factors expressed in *P. pastoris* KM71H. Figure S2: Sodium dodecyl sulfate polyacrylamide gel electrophoresis (SDS-PAGE) of purified VEGF-A_165_ and FGF-2M. Figure S3: Metabolic activity of ADSCs and HUVECs in media containing VEGF-A_165_ or FGF-2M. Figure S4: Influence of FGF-2 on the growth of human and porcine adipose tissue-derived stem cells. Figure S5: Metabolic activity of ADSCs and HUVECs cultivated in wells coated with FGF-2M or VEGF-A_165_. Figure S6: The initial adhesion of ADSCs and HUVECs to wells coated with FGF-2M or VEGF-A_165_.

## Abbreviations

ABAM	Antibiotic antimycotic solution
ADSC	Adipose tissue-derived stem cell
BMGH	Buffered minimal glycerol medium
BMMH	Buffered minimal methanol medium
BMGY	Buffered glycerol complex medium
BMMY	Buffered methanol complex medium
BSA	Bovine serum albumin
DAPI	2-(4-Amidinophenyl)-1*H*-indole-6-carboxamidine
DMEM	Dulbecco’s modified Eagle’s medium
EBM2	Endothelial cell basal medium 2
EGF	Epidermal growth factor
EGM2	Endothelial cell growth medium 2
FBS	Fetal bovine serum
FGF-2	Fibroblast growth factor 2
FGF-2M	Fibroblast growth factor 2 double mutant
HEK	Human embryonic kidney
HUVEC	Human umbilical vein endothelial cell
IGF-1	Insulin-like growth factor 1
MTS	3-(4,5-Dimethylthiazol-2-yl)-5-(3-carboxymethoxyphenyl)-2-(4-sulfophenyl)-2*H*-tetrazolium
MTT	3-[4,5-Dimethylthiazol-2-yl]-2,5-diphenyltetrazolium bromide
PBS	Phosphate-buffered saline
PrADSC	Porcine adipose tissue-derived stem cell
SDS-PAGE	Sodium dodecyl sulfate-polyacrylamide gel electrophoresis
TRITC	Tetramethylrhodamine
VEGF	Vascular endothelial growth factor
VEGF-A	Vascular endothelial growth factor A
VEGFR2	Vascular endothelial growth factor receptor 2
WST	Water-soluble tetrazolium salt
XTT	2,3-*bis*-(2-Methoxy-4-nitro-5-sulfophenyl)-2*H*-tetrazolium-5-carboxanilide
YPD	Yeast extract peptone dextrose
