# Supplementary material for: Growth Factors VEGF-A165 and FGF-2 as Multifunctional Biomolecules Governing Cell Adhesion and Proliferation
Source: Int J Mol Sci. 2021 Feb 12;22(4):1843. doi: 10.3390/ijms22041843 (PMC7917819; doi:10.3390/ijms22041843)
Supplement: Supplementary file 1 [file ijms-22-01843-s001.pdf]

International Journal of *Molecular Sciences*

MDPI

*Supplementary Material*

# Growth Factors VEGF-A$_{165}$ and FGF-2 as Multifunctional Biomolecules Governing Cell Adhesion and Proliferation

**Antonín Sedlář [1,2], Martina Trávníčková [1], Roman Matějka [1,3], Šimon Pražák [1,3], Zuzana Mészáros [4,5], Pavla Bojarová [3,4], Lucie Bačáková [1,*], Vladimír Křen [4] and Kristýna Slámová [4,*]**

1 Laboratory of Biomaterials and Tissue Engineering, Institute of Physiology of the Czech Academy of Sciences, Vídeňská 1083, Praha 4, CZ 14220, Czech Republic; Antonin.Sedlar@fgu.cas.cz (A.S.), Roman.Matejka@fgu.cas.cz (R.M.), Martina.Travnickova@fgu.cas.cz (M.T.), simon.prazak@fgu.cas.cz; simon.prazak@fbmi.cvut.cz (Š.P.), Lucie.Bacakova@fgu.cas.cz (L.B.)
2 Department of Physiology, Faculty of Science, Charles University, Viničná 7, Praha 2, CZ 12844, Czech Republic.
3 Faculty of Biomedical Engineering, Czech Technical University in Prague, CZ 27201 Kladno, Czech Republic.
4 Laboratory of Biotransformation, Institute of Microbiology of the Czech Academy of Sciences, Vídeňská 1083, Praha 4, CZ 14220, Czech Republic; zuzana.meszaros@biomed.cas.cz (Z.M.), bojarova@biomed.cas.cz (P.B.), kren@biomed.cas.cz (V.K.), slamova@biomed.cas.cz (K.S.)
5 Department of Biochemistry, University of Chemistry and Technology Prague, Technická 6, Praha 6, CZ 16628, Czech Republic.
* Correspondence: slamova@biomed.cas.cz; Tel.: +420-296442766 (K.S.); Lucie.Bacakova@fgu.cas.cz; Tel.: +420-296443743 (L.B.)

## List of contents:

1. Amino acid and nucleotide sequences of growth factors expressed in *P. pastoris* KM71H
2. SDS-PAGE of purified VEGF-A$_{165}$ and FGF-2M
3. Metabolic activity of ADSCs and HUVECs in media containing VEGF-A$_{165}$ or FGF-2M
4. Influence of FGF-2 on the growth of human and porcine adipose tissue-derived stem cells
5. Metabolic activity of ADSCs and HUVECs cultivated in wells coated with FGF-2M or VEGF-A$_{165}$
6. The initial adhesion of ADSCs and HUVECs to wells coated with FGF-2M or VEGF-A$_{165}$

## 1. Amino acid and nucleotide sequences of growth factors expressed in *P. pastoris* KM71H

Optional *N*-terminal substrate sequence for factor XIIIa (NQEQVSPL) is shown in blue.

### Amino acid sequence of VEGF-A165

(NQEQVSPL)APMAEGGGQNHHEVVKFMDVYQRSYCHPIETLVDIFQEYPDEIEYIFKPSCVPLMRCGGCCNDEGLE
CVPTEESNITMQIMRIKPHQGQHIGEMSFLQHNKCECRPKKDRARQENPCGPCSERRKHLFVQDPQTCKCSCKNTD
SRCKARQLELNERTCRCDKPRR

### Nucleotide sequence of VEGF-A165 optimized for expression in *P. pastoris*

(AACCAAGAACAAGTTTCACCTCTT)GCTCCAATGGCTGAAGGAGGAGGTCAAAACCATCATGAAGTTGTTAA
GTTTATGGATGTTTACCAAAGATCATACTGTCATCCTATTGAAACTTTGGTTGATATTTTTCAAGAATACCCTG
ATGAAATTGAATACATTTTTAAGCCATCCTGTGTTCCTTTGATGAGATGTGGTGGTTGTTGTAACGATGAAGGA
TTGGAATGTGTTCCTACTGAAGAATCTAACATTACTATGCAAATTATGAGAATTAAGCCTCATCAAGGTCAAC
ATATTGGTGAAATGTCCTTTTTGCAACATAACAAGTGTGAATGTAGACCTAAGAAGGATAGAGCTAGACAAG
AAAACCCATGTGGACCTTGTTCCGAAAGAAGAAAGCATTTGTTTGTTCAAGATCCACAAACTTGTAAGTGTTC
CTGTAAGAACACTGATTCCAGATGTAAGGCTAGACAATTGGAATTGAACGAAAGAACTTGTAGATGTGATAA
GCCTAGAAGA

### Amino acid sequence of FGF-2M (R31K/R129K)

(NQEQVSPL)MAAGSITTLPALPEDGGSGAFPPGHFKDPK**K**LYCKNGGFFLRIHPDGRVDGVREKSDPHIKLQLQAEE
RGVVSIKGVCANRYLAMKEDGRLLASKCVTDECFFFERLESNNYNTYRSRKYTSWYVALK**K**TGQYKLGSKTGPGQK
AILFLPMSAKS

### Nucleotide sequence of FGF-2M optimized for expression in *P. pastoris*

(AACCAAGAACAAGTTTCACCTCTT)ATGGCTGCTGGTTCAATTACTACTTTGCCAGCTTTGCCTGAAGATGGA
GGTTCCGGAGCTTTTCCTCCAGGACATTTTAAAGATCCTAAGAAGTTGTACTGTAAGAACGGTGGATTTTTTTT
GAGAATCCATCCTGATGGTAGAGTTGATGGAGTTAGAGAAAAGTCCGATCCACATATTAAGTTGCAATTGCA
AGCTGAAGAAAGAGGTGTTGTTTCTATTAAGGGAGTTTGTGCTAACAGATACTTGGCTATGAAGGAAGATGG
TAGATTGTTGGCTTCCAAGTGTGTTACTGATGAATGTTTTTTTTTTGAAAGATTGGAATCTAACAACTACAACA
CTTACAGATCCCGTAAGTACACTTCATGGTACGTTGCTTTGAAGAAGACTGGTCAATACAAGTTGGGTTCCAA
GACTGGACCAGGTCAAAAGGCTATTTTGTTTTTGCCTATGTCCGCTAAGTCA

### 2. SDS-PAGE of purified VEGF-A$_{165}$ and FGF-2M

15% SDS-PAGE: LMW Low Molecular Weight Marker (MW of the individual standards are given at the corresponding bands). Red rectangles indicate the position of the respective growth factors (theoretical MW corresponding to amino acid sequences: VEGF-A$_{165}$ 19.16 kDa; FGF-2M 17.20 kDa).

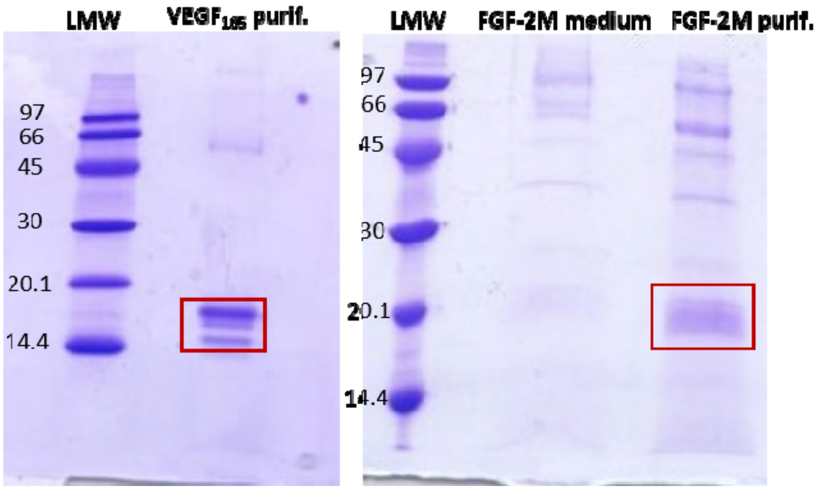


**Figure S1.** SDS-PAGE of purified VEGF-A$_{165}$ and FGF-2M. The red rectangles indicate the positions of the protein bands taken into calculation of the percentual proportions of the respective growth factors in solution by ImageJ software. The multiple bands of VEGF-A$_{165}$ probably represent different *O*-glycosylation variants of the protein.

### 3. Metabolic activity of ADSCs and HUVECs in media containing VEGF-A$_{165}$ or FGF-2M

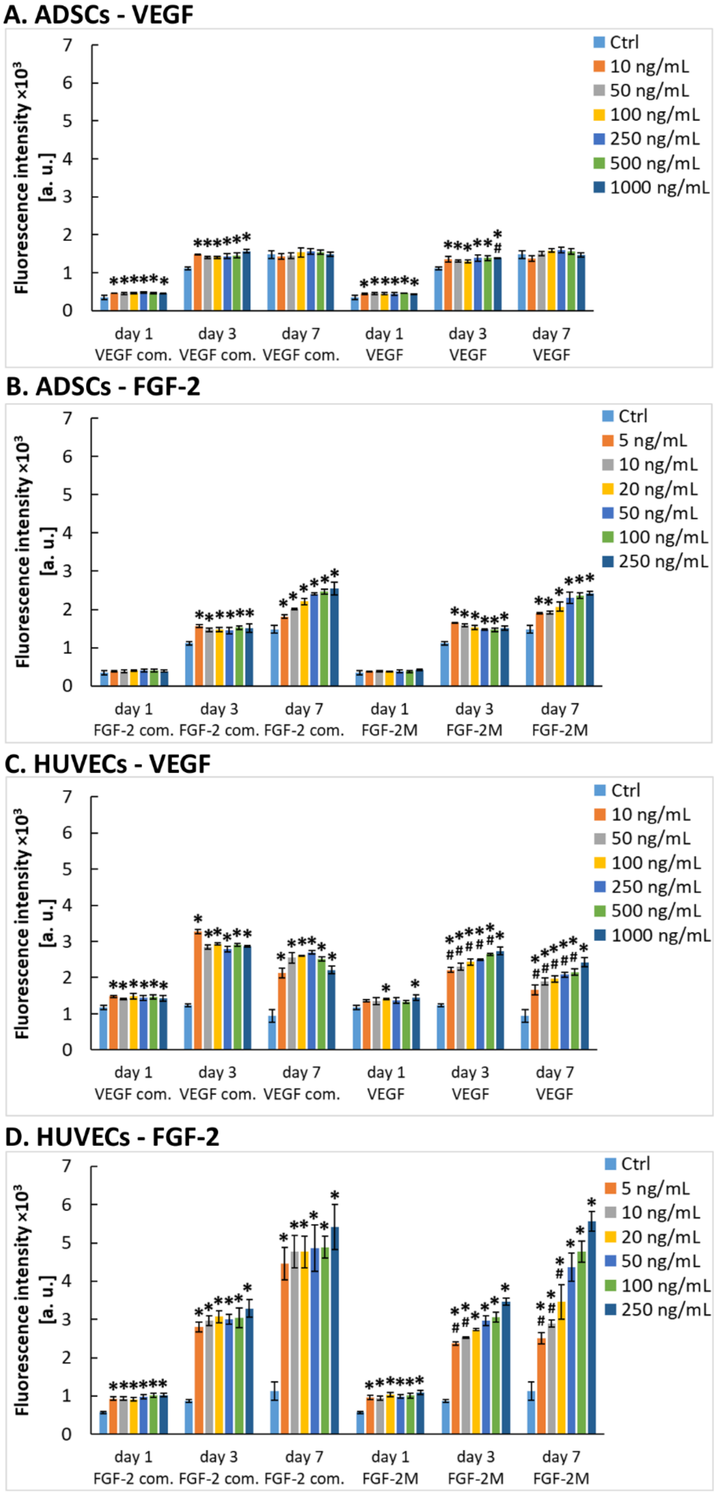


**Figure S2.** Metabolic activity of ADSCs (**A, B**) and HUVECs (**C, D**) grown in media supplemented with VEGF-A165 (**A, C**) or FGF-2M (**B, D**). The cells were grown in media enriched with commercial VEGF-A165 (VEGF com.) or our recombinant VEGF-A165 in concentrations from 10 to 1000 ng/mL (**A, C**), or in media enriched with commercial FGF-2 (FGF-2 com.) or our recombinant FGF-2M in concentrations from 5 to 250 ng/mL (**B, D**). The growth factors were added into DMEM with 10% FBS for ADSCs, and into EGM2-weak for HUVECs. Control cells were grown in media without growth factors (Ctrl). The metabolic activity was determined on days 1, 3, and 7 after cell seeding by a resazurin test. Mean ± SD from 3 wells. Holm-Sidak method, $p \leq 0.05$. Statistically significant differences are depicted above the columns. The samples were statistically compared on the

indicated day after seeding. * - statistically significant difference versus control sample (Ctrl). # - statistically significant difference versus sample containing the corresponding concentration of commercial growth factor.

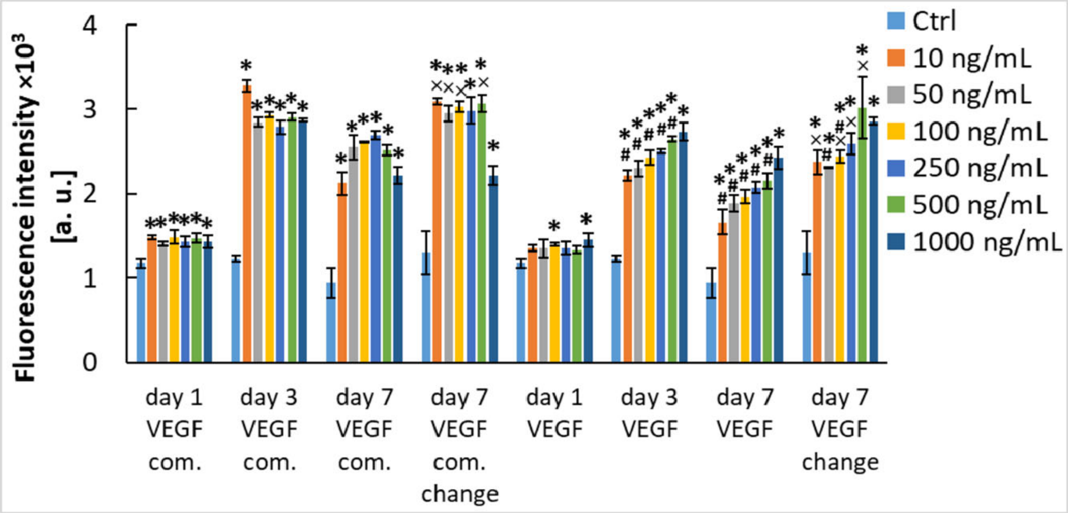


**Figure S3.** The effect of exchange of a medium with VEGF-A165 on the metabolic activity of HUVECs. The HUVECs were grown in media enriched with commercial VEGF-A165 (VEGF com.) or our recombinant VEGF-A165 in concentration range from 10 to 1000 ng/mL. VEGF-A165 was added into EGM2-weak. Control cells were grown in media without growth factors (Ctrl). The cell metabolic activity was determined on days 1, 3, and 7 after seeding by a resazurin assay. In some of the samples, the medium containing the corresponding concentration of growth factor was exchanged for a fresh one on day 3 after cell seeding. Mean ± SD from 3 wells. Holm-Sidak method, $p \leq 0.05$. The samples were statistically compared on the indicated day after seeding. Statistically significant differences are depicted above the columns. * - statistically significant difference versus control sample (Ctrl). # - statistically significant difference versus sample containing corresponding concentration of commercial growth factor. × - statistically significant difference versus sample containing corresponding concentration of growth factor without medium exchange.

# 4. Influence of FGF-2 on the growth of human and porcine adipose tissue-derived stem cells

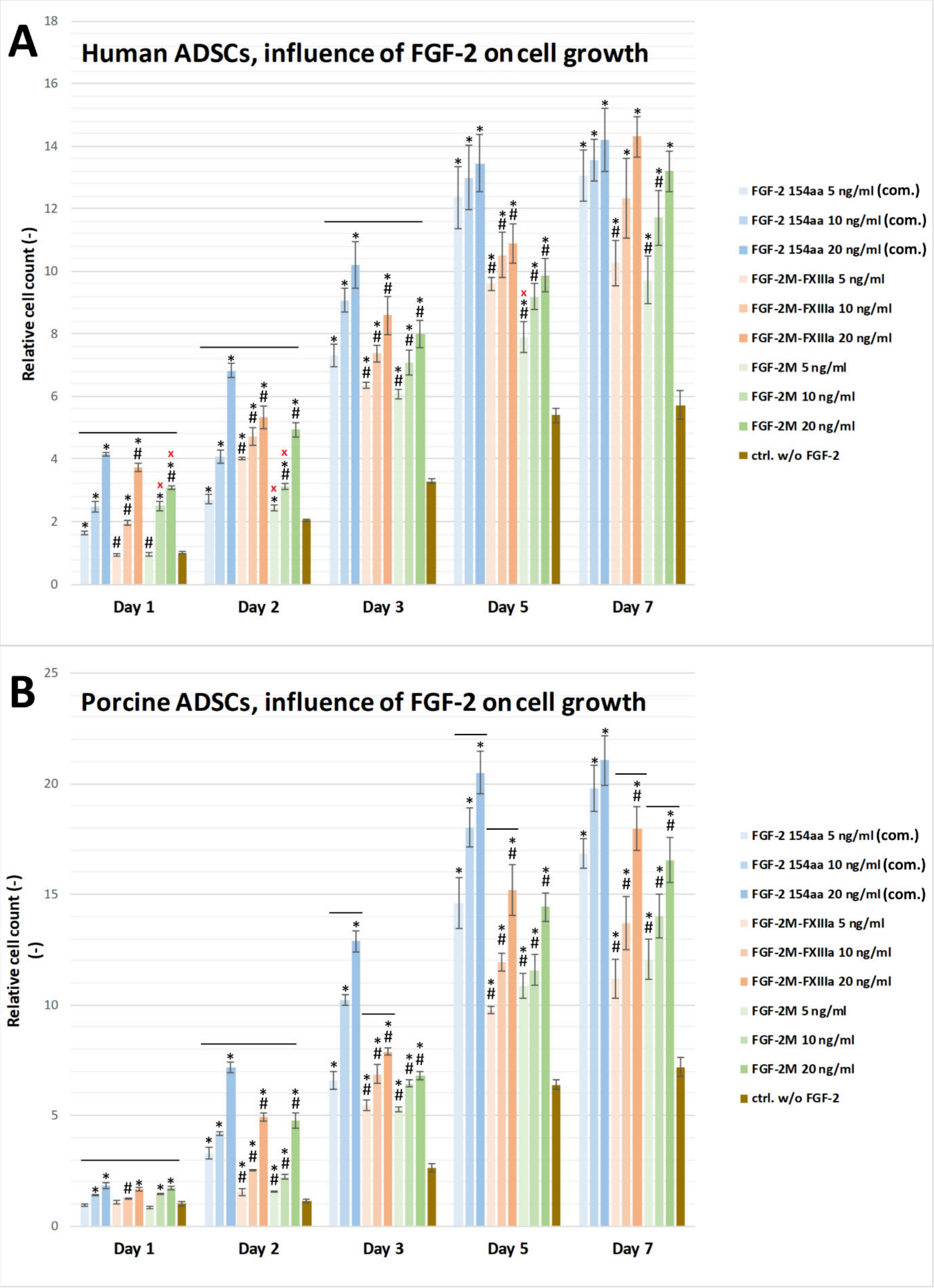


**Figure S4.** Influence of 3 types of FGF-2 with 3 different concentrations on the growth of human (A) and porcine (B) ADSCs. Cell growth is normalized to the 1st-day control sample (set as 1). Cell number is determined on days 1, 2, 3, 5,

and 7. Mean ± SD from 10 random fields of view. Nonparametric Kruskal-Wallis One Way Analysis of Variance on Ranks, Dunn's Method, MATLAB, $p \leq 0.05$. Statistical significance: * - in comparison with control medium without FGF-2; # - in comparison with medium with the corresponding concentration of commercial FGF-2; x – in comparison with the corresponding concentration of FGF-2M-FXIIIa; _______ - statistically significant differences among the concentrations.

## 5. Metabolic activity of ADSCs and HUVECs cultivated in wells pre-adsorbed with VEGF-A165 or FGF-2M

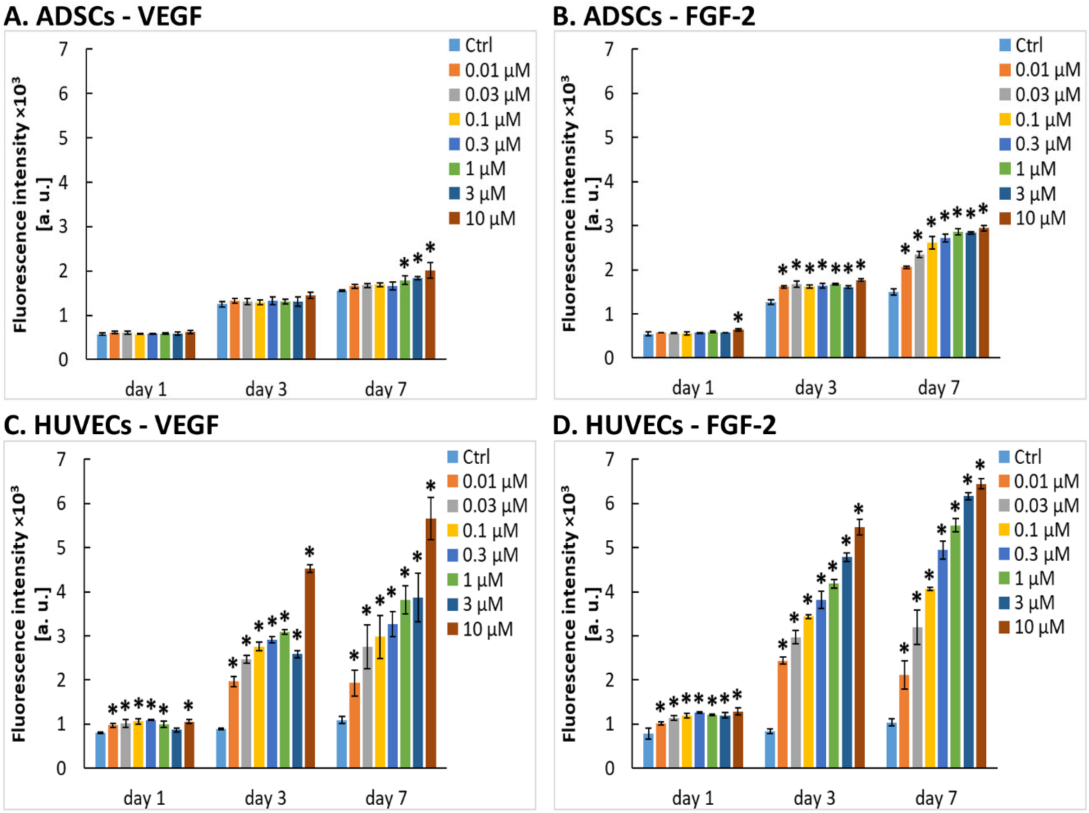


**Figure S5.** The metabolic activity of ADSCs (**A, B**) and HUVECs (**C, D**) in wells of 96-well polystyrene tissue culture plates pre-adsorbed with VEGF-A165 (**A, C**) or FGF-2M (**B, D**) in concentrations from 0.01 to 10 µM. Pristine wells without adsorbed growth factors served as control substrates (Ctrl). ADSCs were grown in DMEM with 10% FBS. HUVECs were grown in EGM2-weak. The metabolic activity was determined on days 1, 3, and 7 after seeding by a resazurin test. Mean ± SD from 3 wells. Holm-Sidak method, $p \leq 0.05$. The samples were statistically compared on the indicated day after seeding. * - a statistically significant difference in comparison with the control sample (Ctrl).

## 6. The initial adhesion of ADSCs and HUVECs to wells coated with VEGF-A$_{165}$ or FGF-2M

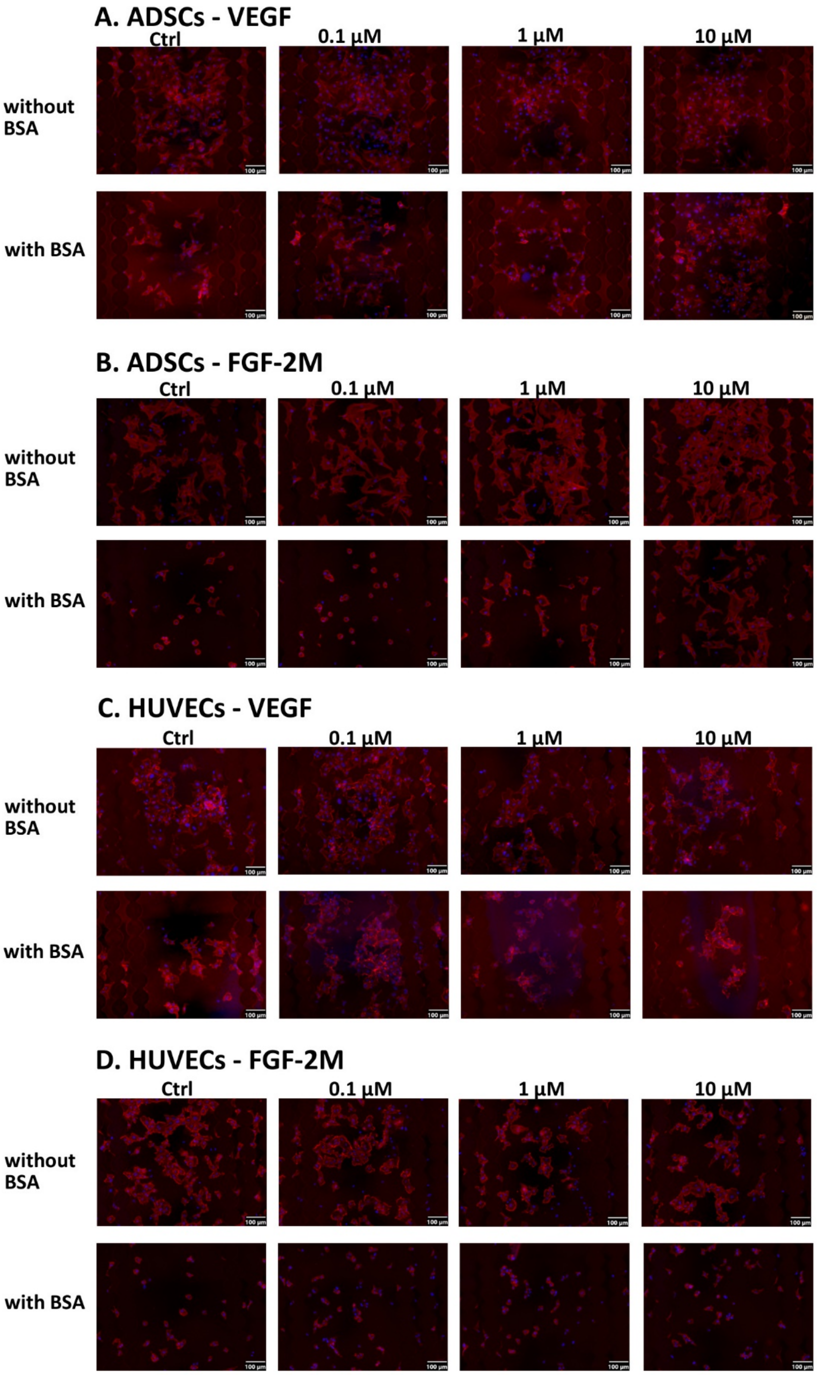


**Figure S6.** Microphotographs of ADSCs (**A, B**) and HUVECs (**C, D**) 4 hours after seeding into wells in 96-well E-plates pre-adsorbed with VEGF-A$_{165}$ (**A, C**) or FGF-2M (**B, D**) in concentrations from 0.01 to 10 μM. The wells either blocked with 0.5% BSA (with BSA) or left unblocked (without BSA). The filamentous actin in cells was stained with phalloidin-TRITC in order to visualize the cell morphology. The nuclei were counterstained with Hoechst 33258. Olympus IX 71 microscope, DP 70 digital camera, obj. 10x, scale bar 100 μm.
